# Identification of Blood Biomarkers for Alzheimer's Disease Through Computational Prediction and Experimental Validation

**DOI:** 10.3389/fneur.2018.01158

**Published:** 2019-01-08

**Authors:** Fang Yao, Kaoyuan Zhang, Yan Zhang, Yi Guo, Aidong Li, Shifeng Xiao, Qiong Liu, Liming Shen, Jiazuan Ni

**Affiliations:** ^1^Shenzhen Key Laboratory of Marine Biotechnology and Ecology, College of Life Science and Oceanography, Shenzhen University, Shenzhen, China; ^2^Key Laboratory of Optoelectronic Devices and Systems of Ministry of Education and Guangdong Province, College of Optoelectronic Engineering, Shenzhen University, Shenzhen, China; ^3^Department of Neurology, Shenzhen People's Hospital, Shenzhen, China; ^4^Department of Rehabilitation, The Eighth Affiliated Hospital of Sun Yat-sen University, Shenzhen, China

**Keywords:** Alzheimer's disease, blood, protein, biomarker, computation

## Abstract

**Background:** Alzheimer's disease (AD) is the major cause of dementia in population aged over 65 years, accounting up to 70% dementia cases. However, validated peripheral biomarkers for AD diagnosis are not available up to present. In this study, we adopted a new strategy of combination of computational prediction and experimental validation to identify blood protein biomarkers for AD.

**Methods:** First, we collected tissue-based gene expression data of AD patients and healthy controls from GEO database. Second, we analyzed these data and identified differentially expressed genes for AD. Third, we applied a blood-secretory protein prediction program on these genes and predicted AD-related proteins in blood. Finally, we collected blood samples of AD patients and healthy controls to validate the potential AD biomarkers by using ELISA experiments and Western blot analyses.

**Results:** A total of 2754 genes were identified to express differentially in brain tissues of AD, among which 296 genes were predicted to encode AD-related blood-secretory proteins. After careful analysis and literature survey on these predicted blood-secretory proteins, ten proteins were considered as potential AD biomarkers, five of which were experimentally verified with significant change in blood samples of AD vs. controls by ELISA, including GSN, BDNF, TIMP1, VLDLR, and APLP2. ROC analyses showed that VLDLR and TIMP1 had excellent performance in distinguishing AD patients from controls (area under the curve, AUC = 0.932 and 0.903, respectively). Further validation of VLDLR and TIMP1 by Western blot analyses has confirmed the results obtained in ELISA experiments.

**Conclusion:** VLDLR and TIMP1 had better discriminative abilities between ADs and controls, and might serve as potential blood biomarkers for AD. To our knowledge, this is the first time to identify blood protein biomarkers for AD through combination of computational prediction and experimental validation. In addition, VLDLR was first reported here as potential blood protein biomarker for AD. Thus, our findings might provide important information for AD diagnosis and therapies.

## Introduction

Alzheimer's disease (AD) is the major cause of dementia in population aged over 65 years, accounting up to 70% dementia cases (1). This disease is pathologically characterized with extracellular senile plaques (amyloid-β, Aβ) and intraneuronal neurofibrillary tangles (NFTs), which are the prime suspects in damaging and killing nerve cells (2). AD has become a major health problem in the world due to the lack of effective treatment. It was reported that there were approximate 48 million people worldwide affected by AD in 2015, and the number was estimated to reach 86 million by the year 2050 (3). Clearly, the increasing AD cases would load great burden on families and society, urging the physicians and scientists to find precise and effective ways to diagnose and treat this disease.

Currently, the clinical diagnosis of AD requires a series of examinations including medical history, neuropsychological assessment, and various radiological investigations (4). However, those diagnosis processes could not be used as routine examinations for AD, because they are time-consuming and largely depend on physician's experience. In order to diagnose AD objectively and accurately, researchers have used biotechnologies and bioinformatics methods to search for disease biomarkers. As cerebrospinal fluid (CSF) is affinity with brain, it is considered to contain potential biomarkers of AD pathologies. Several studies have indicated that the decreased concentration of Aβ_42_ peptide and increased concentration of tau proteins in CSF of AD patients compared to controls might work as diagnostic biomarkers for AD (5, 6). While CSF collection by lumbar puncture is invasive and may lead to some side effects such as headache (7), which limits the application of these biomarkers for large-scale AD screening. Blood contains large number of disease-associated proteins and its obtaining is non-invasive, thus it becomes a good source for discovery of AD biomarkers.

Extensive researches have been done to discover plasma or serum biomarkers for AD. For example, Ray and colleagues used antibody arrays to identify an 18-panel protein signature from 120 cell-signaling proteins, which could differentiate ADs from non-demented controls and could also distinguish mild cognition impairment (MCI) patients who later progressed to AD from those unchanged or converted to other dementia (8). Liao and colleagues recognized 6 possible plasma biomarkers for AD patients by combining 2D-PAGE and LC-MS/MS methods (9). Pratico‘ et al disclosed that the F2-IsoPs, resulting from peroxidation of poly-unsaturated fatty acid (10), have high levels in plasma of AD and MCI patients by using GC-MS technology (11, 12). However, the identified AD biomarkers are discrepant dramatically due to the variations in research methods. Generally, discovery of blood biomarkers for disease was conducted through comparing the proteome of blood samples from disease and control. But this no-targeted method is very challenging because there are lots of proteins with relatively low abundance or with a wide range of orders of magnitude in blood, which could not all be covered by one mass spectrometer (13). As of today, there are no valid biomarkers for AD diagnosis in blood.

In this study, we conducted a combination of computational prediction and experimental validation to identify potential blood protein biomarkers for AD. We firstly analyzed previously published gene expression data of brain tissues from AD patients to identify differentially expressed genes for AD. Furthermore, we applied a blood-secretory protein prediction program on these genes to predict AD-related proteins in blood. Finally, several potential blood protein biomarkers for AD were selected and verified by enzyme-linked immunosorbent assay (ELISA) experiments and Western blot analyses on blood samples from AD patients and healthy controls. This work provides a more specific and effective way to investigate blood protein biomarkers for AD.

## Materials and Methods

The schematic diagram of the workflow in this study was given as Figure S1.

### Gene Expression Data of Brain Tissues From AD Patients

Brain tissue-based gene expression data of AD patients were collected from GEO database (14). Two series of datasets, GSE48350 (15, 16) and GSE5281 (17), were selected for data analyses according to the criteria described as follows: first, the datasets we used for analysis are gene expression data of brain tissues from AD patients and healthy controls; second, each dataset must contain both samples of AD patients and healthy controls; third, the number of AD samples and healthy controls are no less than 10 respectively in each dataset. After analysis, we found that these two datasets meet our screening criteria, and have a relatively large number of samples for data analysis. The two datasets are all generated from the platform of Affymetrix Human Genome U133 Plus 2.0 Array, which includes 43285 probes corresponding to 21246 genes. There are 253 samples (80 ADs and 173 controls) in GSE48350, and 161 samples (87 ADs and 74 controls) in GSE5281. All CEL files of each dataset were downloaded from the database, and normalized by using Robust Multi-array Averaging (RMA) method (18) for further analysis. Detailed information about these samples can be accessed from GEO database.

### Identification of Differentially Expressed Genes for AD

We first identified differentially expressed probes (DEPs), and then mapped these probes to their genes. The following procedure was used to identify DEPs for each dataset. Kolmogorov–Smirnov test (19) was used to examine whether the data come from a normal distribution. If they were from normal distribution, Student's *t*-test would be used to detect DEPs. However, our results showed that the values of many examined probes did not fit normal distribution, Wilcoxon rank sum test (20) was applied to identify DEPs for AD with *p*-value < 0.05 as cutoff for significance. Additionally, Benjamini and Hochberg (21) method was used to control the false discovery rate (FDR) of the selected DEPs with *q*-value < 0.05 as cutoff. In order to further determine which probes were up-regulated and down-regulated in ADs, fold change (FC) was computed across samples for each probe. As a whole, probes with *q*-value < 0.05 and FC > 1.2 were considered up-regulated, and those with *q*-value < 0.05 and FC < 0.833 were down-regulated. Finally, we chose the differentially expressed probes with consistent change trend in these two datasets to map to their corresponding genes, which were considered to be differentially expressed genes for AD.

### Prediction of AD-Related Blood Proteins Based on Differentially Expressed Genes

All differentially expressed genes were analyzed for prediction whether their protein products could be secreted into blood through a program developed by Juan Cui et al (22). The basic idea of this program was summarized as follows. First, human proteins that are known to be secretory proteins and can be detected in plasma/serum due to various pathological conditions were collected to form positive dataset. Second, non-blood-secretory proteins, which include proteins unrelated to secretory pathway and secreted proteins not involved in the circulatory system, were selected as negative dataset. Third, these proteins' physical and chemical properties, amino acid sequence and structural features were collected to identify what these blood-secretory proteins have in common. Fourth, a list of protein features such as signal peptides, glycosylation sites, secondary structural content, hydrophobicity and polarity measures etc. was identified due to their great power in distinguishing blood-secretory proteins from those that were deemed not. Finally, a classifier based on support vector machines (SVM) (23) was constructed to predict the blood-secretory proteins by using the positive and negative datasets and the identified protein features.

### Validation of Potential Blood Protein Biomarkers of AD by ELISA Experiments

In this work, ELISA experiments were carried out on blood samples from AD patients and healthy individuals to validate the predicted blood protein biomarkers for AD. The research protocol of this study was approved by the Human Research Ethics Committee of Shenzhen University and had been performed in accordance with the ethical standards. A total of 123 subjects were enrolled in experiment from Shenzhen People's Hospital and the Eighth Affiliated Hospital of Sun Yat-sen University, including 54 AD patients and 69 healthy subjects. Informed consents were obtained from all participants in accordance with the Declaration of Helsinki prior to their inclusion in this study. All the patients were diagnosed by neuropsychiatrists in the hospital according to the criteria of Diagnostic and Statistical Manual of Mental Disorders-Fourth Edition (DSM-IV). The average age of the patients and controls were 74.3 (ranged from 52 to 93) and 73.9 (ranged from 53 to 94), respectively. The ratio of male to female was about 2:3. In each ELISA experiment, blood samples were selected from AD patients and age- and gender-matched healthy controls. Blood samples (5 ml) were collected using glass tubes. Serums were separated by centrifugation at 3000 g for 10 min, and then subdivided into aliquots and stored at −80°C for further use.

For ELISA experiments, commercial ELISA kits for proteins gelsolin (GSN), brain-derived neurotrophic factor (BDNF), metalloproteinase inhibitor 1 (TIMP1), pigment epithelium-derived factor (SERPINF1) and amyloid-like protein 2 (APLP2) were bought from Uscn Life Science Inc. (Wuhan, China). The catalog numbers of these ELISA kits were SEA372Hu, SEA011Hu, SEA552Hu, SEB972Hu, and SEG122Hu, respectively. Additionally, ELISA kits of inositol 1,4,5-trisphosphate receptor-interacting protein (ITPRIP), transmembrane emp24 domain-containing protein 10 (TMED10), very low-density lipoprotein receptor (VLDLR), mitogen-activated protein kinase 8 (MAPK8) and mitogen-activated protein kinase 1 (MAPK1) were bought from Sbj Biological technology Co., Ltd. (Nanjing, China) with catalog numbers of SBJ-H2157, SBJ-H2158, SBJ-H1100, SBJ-H2160, and SBJ-H2161, respectively. The concentrations of these proteins were measured under the manufacturer's instructions. The total protein concentrations of samples were determined using bicinchoninic acid (BCA) protein assay kit with product No. 23227 (Beyotime, Jiangsu, China).

### Statistical Analyses for ELISA Experiments

Protein concentration of each sample detected by ELISA was normalized with its total protein concentration. For the normalized protein concentrations, G-test (24) was applied to detect the outliers for each group. Software GraphPad Prism 5 was used to visualize the normalized protein concentrations of AD samples and healthy controls. *T*-test was applied to make differential analysis on normalized protein concentrations of AD samples vs. controls, and then FDR (21) was employed to adjust the *p*-values obtained from *T*-test, using 0.05 as significant cutoff. Furthermore, receiver operating characteristic (ROC) curve analysis was carried out to evaluate the power of these proteins in distinguishing AD samples from healthy controls, which was generated by using package pROC on R (25, 26).

### Further Validation of the Potential Protein Biomarkers of AD by Western Blot Analyses

To further validate the potential protein biomarkers of AD in blood, Western blot analyses were carried out on un-depleted serum samples of AD patients and healthy controls by specific antibodies. Total protein concentrations of these samples were measured by the BCA assay. Proteins (10 μg) were separated by SDS-PAGE on 12% polyacrylamide gels. After electrophoresis, the proteins were transferred onto 0.2 μm polyvinylidene fluoride (PVDF) membranes (Millipore, Massachusetts, USA), and the membranes were blocked with 5% nonfat-dried milk in Tris-buffered saline (TBS: 100 mM Tris, and 1.5 M NaCl, pH 7.6) for 1 h and then washed with TBS containing 0.4% (v/v) tween 20 (TBST), followed by incubation with primary antibodies (Bioss Biotechnology, Beijing, China) against VLDLR and TIMP1 overnight at 4°C and horseradish peroxidase (HRP)-conjugated secondary antibody (1:8000, Abmart Inc, Shanghai, China) for 2 h at room temperature. The membranes were washed three times each for 10 min in TBST and developed with enhanced chemiluminescence (ECL) kit (FDbio-Femto ECL kit, FDbio Science Biotech co., Ltd, Hangzhou, China). Immunoreactive signals were detected using a Kodak Image Station 4000M imaging system (Carestream Health Inc., Rochester, NY, USA). Quantitative analysis was performed on the protein bands by ImageJ analysis software (National Institutes of Health, USA). Equal amount of proteins were separated by SDS-PAGE and stained with Coomassie blue, which was used as the loading control.

### Statistical Analysis for Western Blot

The data of Western blot were analyzed using the two-tailed Student's *t*-test to examine any significant differences between ADs and controls by GraphPad Prism 7 software (GraphPad Software, USA) and presented as the means ± the standard errors of the means (SEM). Differences were considered significant with *p*-value < 0.05.

## Results

### Identification of Differentially Expressed Genes in the Brain Tissues of AD Patients

Two brain tissue-based gene expression datasets of AD patients were downloaded from GEO database. There were 5481 DEPs (2511 up-regulated and 2970 down-regulated) identified in GSE48350 and 12115 DEPs (4675 up-regulated and 7440 down-regulated) in GSE5281. Further comparing analysis was made on these two groups of DEPs, and 1545 probes (corresponding to 1186 genes) and 1981 probes (corresponding to 1568 genes) were found consistently up- and down-regulated in these two datasets, respectively (27). In addition, pathway enrichment analysis was conducted on these genes and showed that focal adhesion, TGF-β signaling pathway, and MAPK signaling pathway were significantly enriched by up-regulated genes, and synapse transmission, neuronal system, and calcium signaling pathway were significantly enriched by down-regulated genes [complete list shown in our previous study (27)]. These pathways are consistent with previous observations that AD is associated with neuronal damage and apoptosis, synaptic dysfunction, neuronal activity alteration, blood brain barrier dysfunction, neuro inflammation, oxidative stress, mitochondrial function and aberrant lipid metabolism (28). Therefore, these differentially expressed genes are speculated to be associated with AD pathogenesis.

### Prediction of AD-Related Protein in Blood

It is well known that blood-brain barrier (BBB) controls substances exchange strictly between brain and blood. However, some evidence indicates that breakdown of BBB may account for AD occurrence or aggravation and could enhance the movement of proteins between brain and blood in either direction (29, 30). Thereby, there might be some protein biomarkers reflecting AD pathology in blood. Based on the information described above, we applied a program developed by Juan Cui et al (22) on the differentially expressed genes of AD to predict whether the corresponding proteins could be secreted into blood. Consequently, a total of 296 proteins encoded by 115 up-regulated and 181 down-regulated genes were predicted to be blood-secretory proteins, suggesting that they might be AD-related proteins in blood (Table S1). Some of these proteins have been previously reported as AD biomarkers, such as gelsolin (31), serotransferrin (32, 33), metalloproteinase inhibitor 1 (34), mitogen-activated protein kinase 1 (35), pigment epithelium-derived factor (36) and brain-derived neurotrophic factor (37, 38).

**Figure 1:**
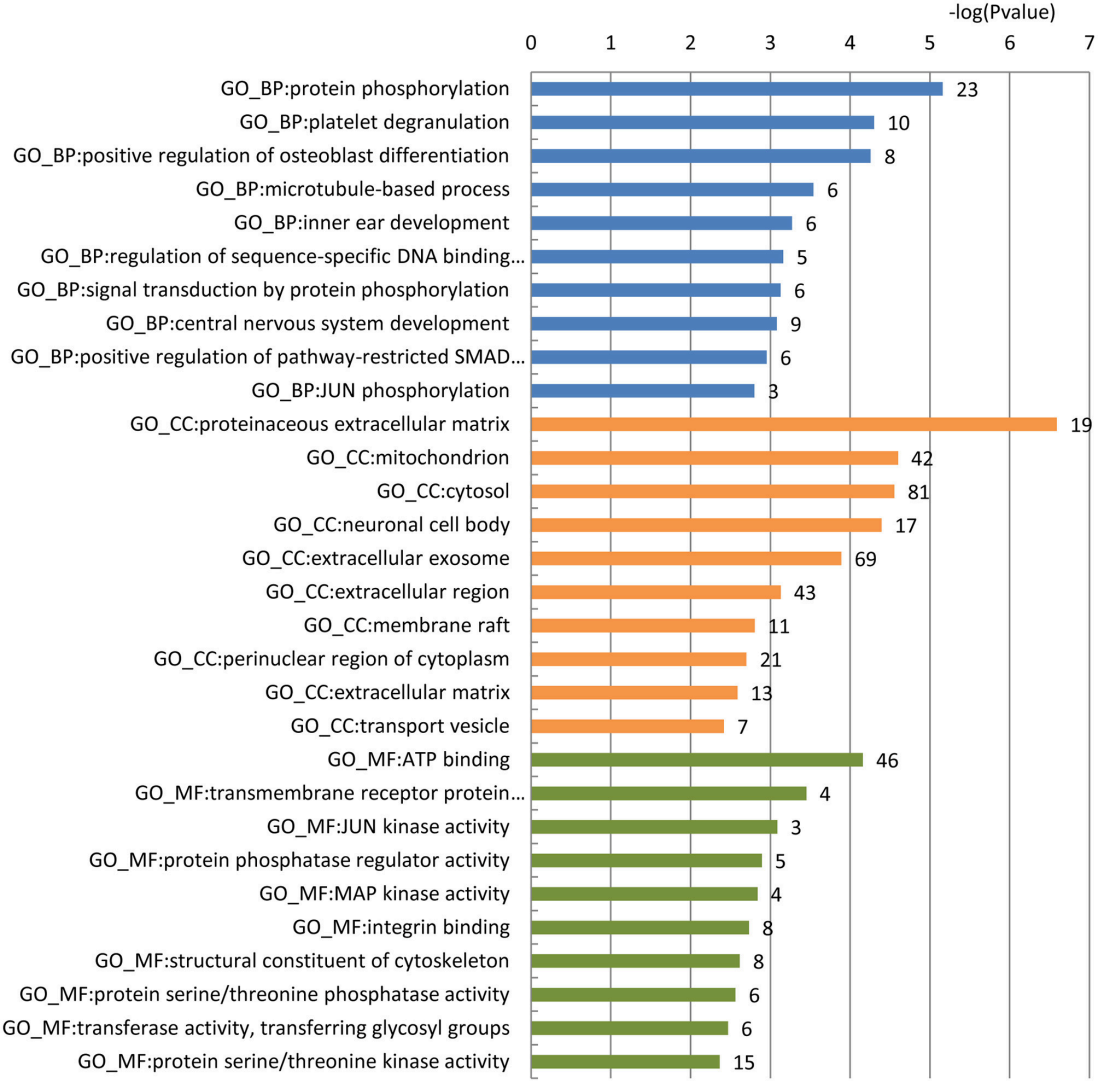
GO enrichment analysis of the 296 predicted blood-secretory proteins. Blue, orange and green bars represent enriched biological processes, cellular components and molecular functions, respectively. The number of proteins enriched in each GO term is shown along with each bar.

To gain a comprehensive understanding of these predicted AD-related blood-secretory proteins, we carried out GO enrichment analysis using DAVID (39). A variety of GO terms were enriched, including 66 biological processes, 30 cellular components and 30 molecular functions (Table S2). We found that the biological processes such as protein phosphorylation and microtubule-based process, cellular components like mitochondrion and neuronal cell body, and molecular functions like ATP binding and MAP kinase activity were enriched, which are all known to be involved in the development of AD. The top 10 GO terms of biological processes, cellular components and molecular functions are shown in Figure 1.

To further choose precise and important candidate biomarkers for AD, we manually checked the relationship between these proteins and AD through database and literature studies. First, we collected a total of 1493 AD-related genes from three databases, 1291 from GAD (40), 169 from KEGG (41), and 197 from MALACARDS (42). Generally, if genes were related with AD, their corresponding protein products were considered to be AD-related as well. Thus, 1493 proteins encoded by these AD-related genes were AD-related proteins. Second, we made literature searches and compiled 167 proteins that have been reported as potential blood biomarkers of AD. Third, we combined the AD-related proteins collected from database and literature, and obtained a total of 1590 AD-related proteins. Finally, we made a comparison analysis between these reported AD-related proteins with 296 predicted blood-secretory proteins, and found that 35 proteins were consistent in these two groups (Table 1).

**Table 1:** The list of 35 AD-related blood-secretory proteins.

**Uniprot ID**	**Protein name**	**Gene name**
P17655	Calpain-2 catalytic subunit	CAPN2
P19438	Tumor necrosis factor receptor superfamily member 1A	TNFRSF1A
P02654	Apolipoprotein C-I	APOC1
P01033	Metalloproteinase inhibitor 1	TIMP1
P02787	Serotransferrin	TF
Q15165	Serum paraoxonase/arylesterase 2	PON2
Q16875	6-phosphofructo-2-kinase/fructose-2,6-bisphosphatase 3	PFKFB3
Q8IWB1	inositol 1,4,5-trisphosphate receptor interacting protein	ITPRIP
Q9UQE7	Structural maintenance of chromosomes protein 3	SMC3
P25774	Cathepsin S	CTSS
P49716	CCAAT/enhancer-binding protein delta	CEBPD
Q9Y2G2	Caspase recruitment domain-containing protein 8	CARD8
P36894	Bone morphogenetic protein receptor type-1A	BMPR1A
P49755	Transmembrane emp24 domain-containing protein 10	TMED10
Q9Y2J8	Protein-arginine deiminase type-2	PADI2
P28482	Mitogen-activated protein kinase 1	MAPK1
P16298	Serine/threonine-protein phosphatase 2B catalytic subunit beta isoform	PPP3CB
P98155	Very low-density lipoprotein receptor	VLDLR
P23560	Brain-derived neurotrophic factor	BDNF
Q00005	Serine/threonine-protein phosphatase 2A 55 kDa regulatory subunit B beta isoform	PPP2R2B
P29120	Neuroendocrine convertase 1	PCSK1
O76003	Glutaredoxin-3	GLRX3
P05019	Insulin-like growth factor I	IGF1
Q01581	Hydroxymethylglutaryl-CoA synthase, cytoplasmic	HMGCS1
Q8NBU5	ATPase family AAA domain-containing protein 1	ATAD1
Q96FJ0	AMSH-like protease	STAMBPL1
O14975	Very long-chain acyl-CoA synthetase	SLC27A2
P02753	Retinol-binding protein 4	RBP4
P40938	Replication factor C subunit 3	RFC3
O00451	GDNF family receptor alpha-2	GFRA2
Q06481	Amyloid-like protein 2	APLP2
P45983	Mitogen-activated protein kinase 8	MAPK8
P53779	Mitogen-activated protein kinase 10	MAPK10
P06396	Gelsolin	GSN
P36955	Pigment epithelium-derived factor	SERPINF1

**Figure 2:**
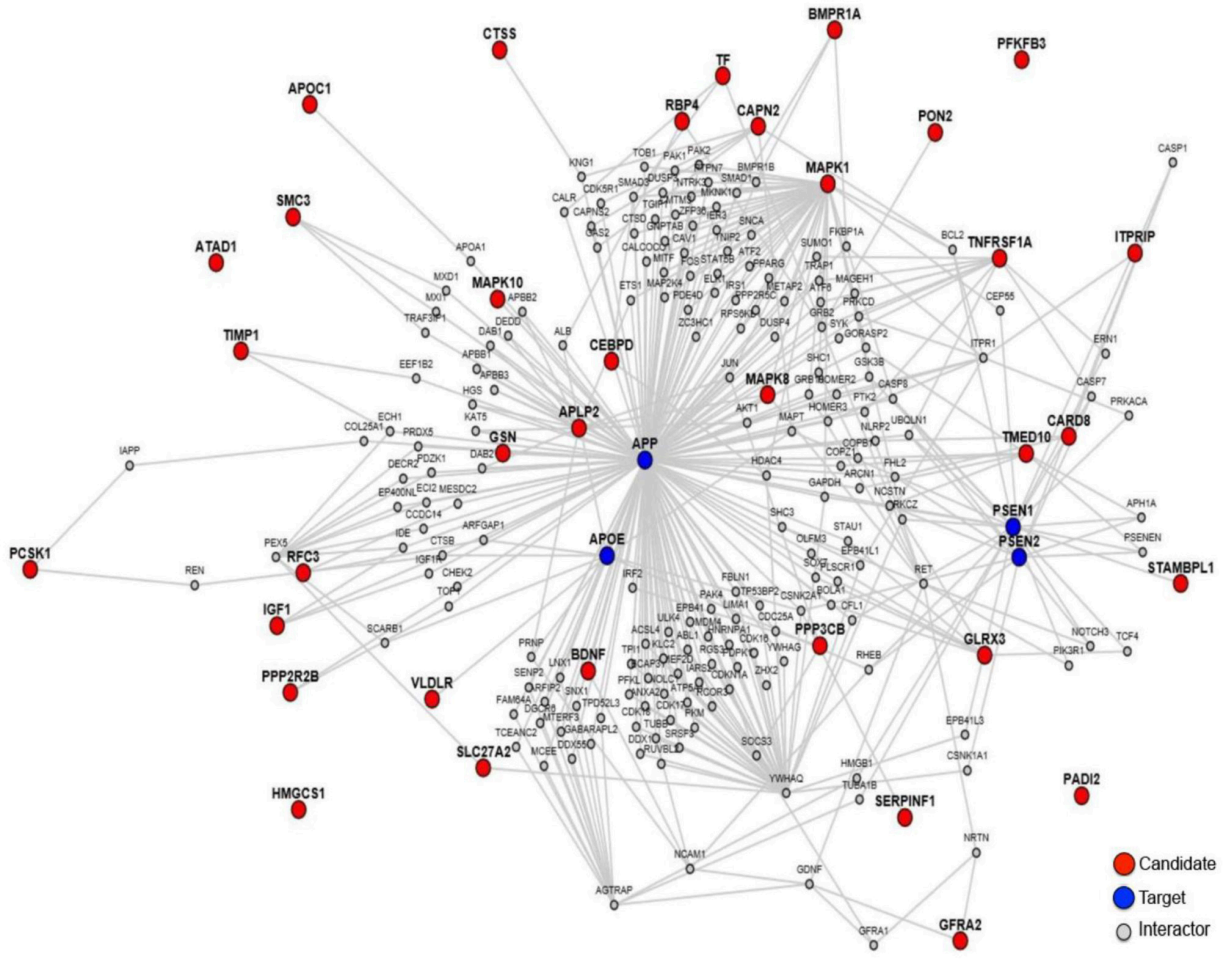
The protein-protein interaction network of the 35 proteins. The red notes are 35 proteins worked as candidates, and the blue notes are AD pathology related proteins worked as targets.

In order to explore the relationship between these 35 proteins and AD pathology, we made a protein-protein interaction analysis through the online sever LENS (43). A network was generated, which contains the 35 AD-related proteins presented by red nodes, 4 key AD pathology related proteins (APP, APOE, PSEN1, and PSEN2) presented by blue nodes and other proteins presented by gray nodes, which connect the 35 proteins with the 4 key proteins (Figure 2). In the network, most proteins are connected to these 4 key proteins except PFKFB3, HMGCS1, ATAD1, and PADI2, suggesting that almost all these proteins were associated with AD pathogenesis.

### Validation of Potential Protein Biomarkers of AD in Blood by ELISA Experiments

Based on the gene expression levels of these 35 proteins in AD samples and their functional annotations, 10 proteins were chosen for experimental verification. They are GSN, BDNF, TIMP1, SERPINF1, ITPRIP, TMED10, VLDLR, MAPK8, APLP2, and MAPK1.

**Figure 3:**
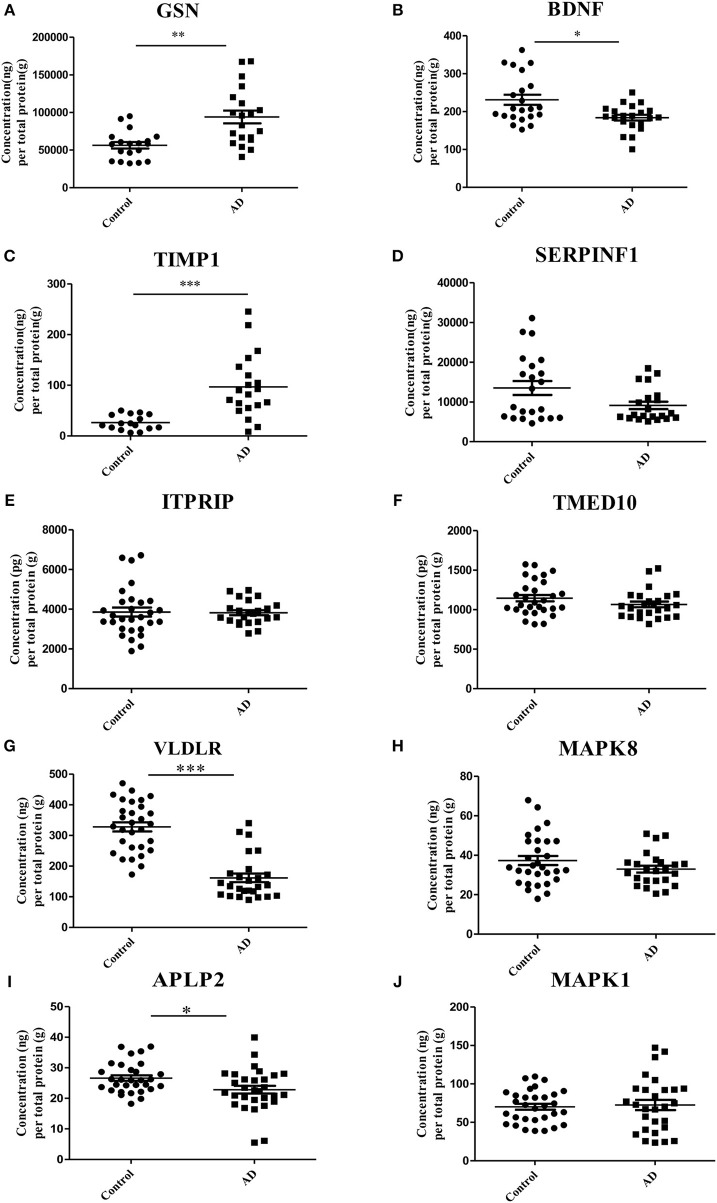
Validation of the 10 selected proteins between AD samples and health controls by ELISA experiment. **(A)** The concentration of protein GSN in serum samples of AD and control. **(B)** The concentration of protein BDNF in serum samples of AD and control. **(C)** The concentration of protein TIMP1 in serum samples of AD and control. **(D)** The concentration of protein SERPINF1 in serum samples of AD and control. **(E)** The concentration of protein ITPRIP in serum samples of AD and control. **(F)** The concentration of protein TMED10 in serum samples of AD and control. **(G)** The concentration of protein VLDLR in serum samples of AD and control. **(H)** The concentration of protein MAPK8 in serum samples of AD and control. **(I)** The concentration of protein APLP2 in serum samples of AD and control. **(J)** The concentration of protein MAPK1 in serum samples of AD and control. *p < 0.05 vs. controls; **p < 0.05 vs. controls; ***p < 0.0005 vs. controls.

ELISA experiments were performed to examine the protein levels in blood samples from AD patients and healthy controls. Figure 3 shows that the expression levels of five proteins were significantly changed in AD samples vs. controls, among which GSN and TIMP1were increased in AD samples, while BDNF, VLDLR and APLP2 were decreased. Furthermore, comparison analyses were carried out on the results of computational prediction and experimental validation (Table 2). We found that these five proteins were consistent in their change trend among prediction and validation. In order to investigate whether age and gender would affect our validation results, further statistical analyses were made on the concentrations of these five proteins according to the different age stages and genders of samples with AD and healthy controls (Figures S2, S3). We found that almost all these five proteins were significantly changed in samples of AD vs. control at different age stages and genders. Even though APLP2 is not changed with statistical significance in samples of AD vs. control at age stage 70–89, and BDNF and APLP2 are not significantly changed in male samples of AD vs. control, they still have downward trend in AD samples compared to controls, indicating that age and gender do not affect our experimental validation results.

**Table 2:** The results of 10 proteins in computational prediction and experimental validation.

**Proteins**	**Computational result**	**Experimental result**						
		**Up/down**	**Means of protein concentrations**		**Means of relative protein concentrations**		***P*-value**	**FDR**
			**Control**	**AD**	**Control**	**AD**	
GSN	Up	Up	3512.06 (ng/ml)	5661.87 (ng/ml)	56470.35 (ng/g)	94026.19 (ng/g)	0.0004	0.0013
BDNF	Down	Down	15.55 (ng/ml)	12.04 (ng/ml)	231.09 (ng/g)	183.97 (ng/g)	0.0042	0.0105
TIMP1	Up	Up	1.65 (ng/ml)	5.18 (ng/ml)	26.49 (ng/g)	96.86 (ng/g)	0.0001	0.0005
SERPINF1	Down	Down	789.96 (ng/ml)	515.23 (ng/ml)	13508.58 (ng/g)	9117.03 (ng/g)	0.0345	0.0575
ITPRIP	Up	–	295.20 (pg/ml)	279.96 (pg/ml)	3855.06 (pg/g)	3825.37 (pg/g)	0.916	0.916
TMED10	Up	–	75.51 (pg/ml)	78.10 (pg/ml)	1145.62 (pg/g)	1066.62 (pg/g)	0.1542	0.1933
VLDLR	Down	Down	26.36 (ng/ml)	11.77 (ng/ml)	327.92 (ng/g)	161.63 (ng/g)	0.0001	0.0005
MAPK8	Down	–	2.48 (ng/ml)	2.41 (ng/ml)	37.33 (ng/g)	32.98 (ng/g)	0.1546	0.1933
APLP2	Down	Down	1.80 (ng/ml)	1.68 (ng/ml)	26.62 (ng/g)	22.83 (ng/g)	0.0184	0.0368
MAPK1	Down	–	5.45 (ng/ml)	5.30 (ng/ml)	70.31 (ng/g)	72.60 (ng/g)	0.7631	0.8479

*In the table, up and down represent up-regulated and down-regulated proteins in the blood samples of AD patients when compared with those of controls*.

**Figure 4:**
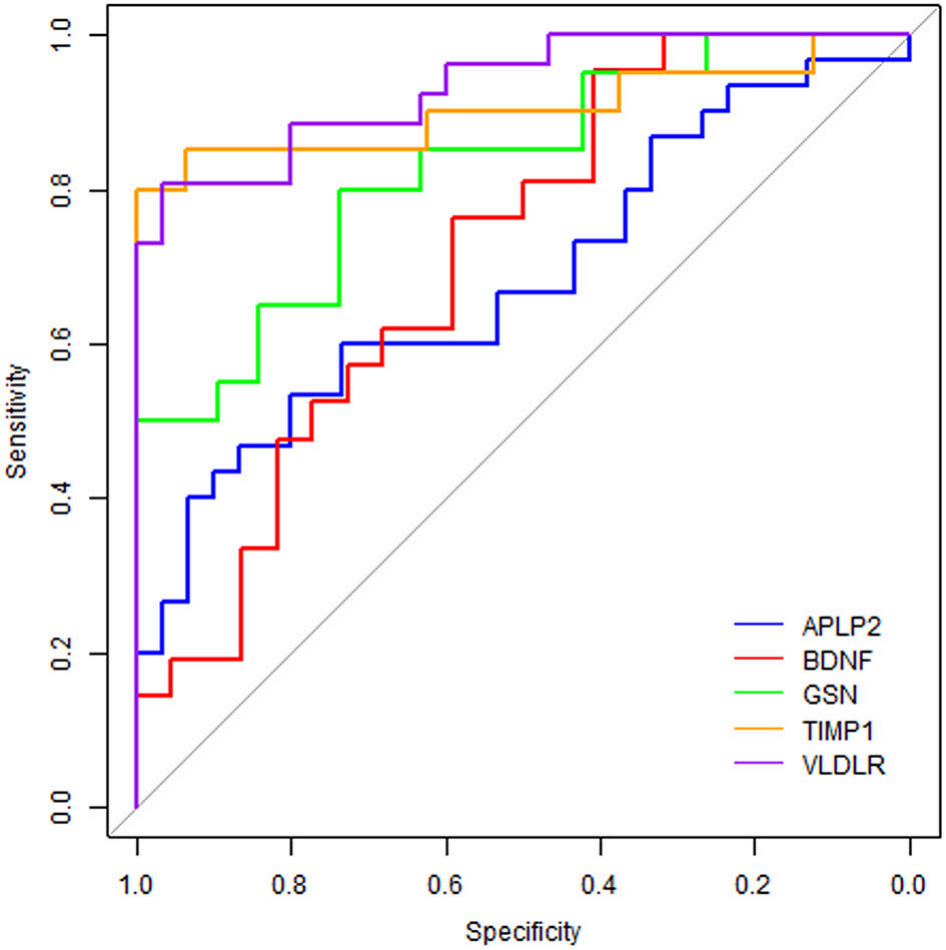
Receiver operating characteristic curve analyses on the 5 proteins. The blue line represents protein APLP2, the red line is BDNF, the green line is GSN, the orange line is TIMP1 and the purple line is VLDLR.

ROC curve analyses were used to evaluate the performance of the five significantly changed proteins in distinguishing AD samples from controls (Figure 4). We found that VLDLR had the most discriminative ability with the area under the curve (AUC) of 0.932 (sensitivity 80.8%, specificity 96.7%), the AUC of TIMP1 was 0.903 (sensitivity 80.0%, specificity 100%) and the AUCs of GSN, BDNF and APLP2 were 0.826, 0.714, and 0.682 respectively. Since VLDLR and TIMP1 were with AUCs larger than 0.85, suggesting that they are more powerful in identifying ADs from controls, and might serve as potential protein biomarkers for AD in blood. Even though the AUCs of GSN, BDNF, and APLP2 were less than 0.85, they could also provide important information for AD diagnosis and therapies.

### Further Validation of Potential Protein Biomarkers for AD by Western Blot Analyses

**Figure 5:**
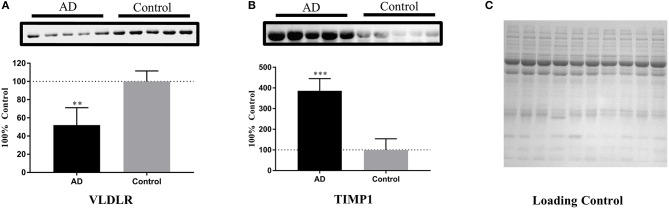
Further validation of the potential protein biomarkers for AD by Western blot analyses. **(A)** The concentration of VLDLR was decreased significantly in AD samples, with **p < 0.01 vs. control samples. **(B)** The concentration of TIMP1 was increased significantly in AD samples, with ***p < 0.001 vs. control samples. The expression level of protein was normalized by the mean of the controls (*n* = 5), with each bar representing SEM. The upper images of Western blot analysis correspond to the lower histograms of semi-quantification. The statistical results of the data were show as *p < 0.05, **p < 0.01, ***p < 0.001. **(C)** A loading control is presented aiming to verify the normalization of protein amounts.

Based on the ELISA analyses, VLDLR and TIMP1 were chosen for further validation of their abilities in identifying the samples of AD patients by Western blot analyses. The serum samples of 5 AD patients and 5 age- and gender-matched healthy controls were used to detect the expression levels of these two proteins. After densitometry analysis on Western blots, VLDLR and TIMP1 were found down- and up-regulated in AD patients respectively as shown in Figure 5, which confirmed the results obtained in the ELISA experiments.

## Discussion

AD is the major cause of dementia. However, there are no valid biomarkers for AD diagnosis in blood so far. In this study, we searched for potential protein biomarkers of AD in blood through computational prediction combined with experimental verification. Based on this strategy, we predicted 296 AD-related blood-secretory proteins, which were predominant enriched in protein phosphorylation, microtubule-based process, mitochondria and MAP kinase activity. As widely known, AD is characterized by neurodegenerative plaques and neurofibrillary tangles in brain (44). Tau protein is microtubule-associated phosphoprotein, whose homeostasis plays a critical role in maintaining the microtubule stability. Hyperphosphorylation of tau has been confirmed to cause dynamic instability and disintegration of microtubule, and then formation of neurofibrillary tangles, which would result in neurodegeneration in the end (45). In addition, reactive oxygen species (ROS) have been reported to involve in the AD pathology mechanisms (46). Mitochondria are the most important places to generate ROS in AD. Some evidence indicated that mitochondria dysfunction in the patients of AD enhanced the oxidative stress and the cellular apoptosis (44). Since these predicted proteins were mainly involved in the processes related to AD pathogenesis (47), we considered that these proteins might be associated with AD pathology.

After careful analyses on these 296 proteins, 10 proteins were chosen for experimental validation by ELISA. Five proteins (GSN, BDNF, TIMP1, VLDLR, and APLP2) were verified to be differentially expressed in AD patients vs. controls, suggesting that they might serve as potential biomarkers for AD in blood. Among them, GSN, BDNF, and TIMP1 have been reported to be potential blood protein biomarkers for AD in previous studies (34, 38, 48, 49), while VLDLR and APLP2 were first time reported here as potential protein biomarkers for AD in blood. To further understand the role of these proteins in the pathogenesis of AD, we present the relationship of these proteins with AD in details in the following parts.

GSN was reported to be implicated in AD due to its level changed with AD progression (50). GSN could bind amyloid beta (Aβ) peptide, inhibit its fibrillization, solubilize reformed Aβ fibrils, and promote its clearance from brain (51). Some studies found that the expression level of GSN was increased in serums of AD compared to controls (49), but others found the decreased expression level of GSN in plasm of AD vs. controls (48). In this study, we predicted and verified that the level of GSN was significantly higher in serums of AD comparing with controls, which was inferred that high expression level of GSN might attribute to the neuroprotective response in AD subjects through immune compensatory system.

BDNF could support the survival of existing neurons and encourage the growth and differentiation of new neurons and synapses (52, 53). Previous studies suggested that BDNF had protective effects on neurons by reducing amyloid beta toxicity (54). BDNF depletion led to an increase in the numbers and size of the cortical amyloid plaque through analyzing on transgenic mouse model of AD (55). It has been reported that BDNF is lower in brain tissue of AD patients (54), which is consistent with our analysis. Kim BY and colleagues made a comprehensive systematic review and meta-analysis on articles and found that BDNF was increased in early AD serum samples and decreased in AD with low MMSE scores respectively comparing with healthy individuals (38). In this study, lower BDNF expression was predicted and experimentally confirmed in blood of AD patients.

TIMP1 is a tissue inhibitor of MMP9 and plays an important role in the development of AD for its function of inflammatory mediation (56). MMP9 was reported to be associated with neurodegeneration processes including extracellular Aβ degradation, neurons degeneration and neurofibrillary tangles formation (57), thus TIMP1 interacting with MMP9 promoted cell proliferation of glial and enhanced the inflammatory response to eliminate amyloid deposition from AD (56). Meanwhile, neurotoxic Aβ fragment could induce the release of MMP9 and TIMP1, and cause their expression changes, which was correlated with the neurotoxicity process (58). The imbalance of levels between MMP9 and TIMP1 in AD patients was associated with senile plaque homoeostasis and tau oligomer formation in brain regions. James D. Doecke and colleagues identified that the level of TIMP1 in plasma of AD was higher than that in healthy controls (34). However, Lorenzl S et al did not observe the level change of TIMP1 in plasma between AD patients and healthy subjects (59). Herein, we found that the level of TIMP1 was significantly up-regulated in AD serums.

VLDLR is an apolipoprotein E receptor involved in synaptic plasticity, learning, and memory (60). It was presented at synaptic compartments, and could alter presynaptic composition and postsynaptic dendritic spine formation through the Ras signaling pathway that is associated in neurodegeneration such as AD (60). Thus, it could be speculated that VLDLR might involve in AD pathogenesis through Ras signaling pathway. Additionally, VLDLR was reported to be one of receptors for AD-related risk factor ApoE (61). ApoE4 was shown to mediate its effects in AD pathogenesis by interfering with Reelin signaling in the brain (62). While Reelin is the major ligand for VLDLR, so it could be speculated that VLDLR might be involved in AD pathogenesis through the ApoE4-Reelin pathway as well. In our study, we found that VLDLR was down-regulated in the brain of AD patients and its encoded protein was predicted and validated with a lower concentration level in blood of AD patients relative to controls.

APLP2, an APP like protein, could bind to synaptic signaling molecules exhibiting synaptogenic activity (63). Furthermore, APLP2 shares essential functions with APP, as it could also interact with proteins Stub1 and CRL4 (CRBN) to facilitate ubiquitination of proteins involved in presynaptic functions and neurodegeneration (64). Herein, we predicted and validated that the encoded protein of APLP2 was down-regulated in the blood of AD patients.

As a whole, this novel biomarker discovery strategy, namely computational prediction combined with experimental verification, provides some potential blood biomarkers for AD. To our knowledge, this is the first report to use such a strategy for AD blood biomarker discovery. Meanwhile, VLDLR is the first time reported here as potential protein biomarker for AD in blood. In addition, this strategy for biomarker discovery could also be used for discovering biomarkers of other nervous system diseases such as Parkinson's disease. Worth noting, this method provides an effective way to find pathology-associated biomarkers in blood, but there are still some shortages in this strategy that could affect our results. For example, there might be some false positive blood-secretory proteins coming from the computational prediction, so the sensitivity of the blood-secretory protein predictor need to be improved in the future. Additionally, gene expression changes in ADs vs. controls could not accurately reflect their proteins' expression changes, so the predicted proteins need to be validated on large scale blood samples further.

## Conclusion

A total of 2754 genes were identified differentially expressed in brain tissues of AD, among which 296 genes were predicted to encode blood-secretory proteins. GO enrichment analysis on the predicted blood-secretory proteins suggested that they were associated with AD and might act as candidate protein biomarkers of AD in blood. Furthermore, ten proteins were chosen for validation by ELISA and five proteins (GSN, BDNF, TIMP1, VLDLR, and APLP2) were validated changed significantly in serum samples of AD vs. controls. ROC curves analyses on these five proteins showed that VLDLR and TIMP1 were with more power in distinguishing AD samples from controls. Western blot analyses on VLDLR and TIMP1 were further revealed that they might serve as potential blood biomarkers for AD. Obviously, further studies are required to confirm these findings.

## Author Contributions

FY, QL, and JN conceived and designed this study. YZ collected data from database and literature. KZ and SX designed and performed the experimental work. FY and LS processed the data and carried out the statistical analysis. YG and AL recruited and diagnosed the patients and provided the blood samples. FY and LS wrote the manuscript. All authors have read and proved the final manuscript.

### Conflict of Interest Statement

The authors declare that the research was conducted in the absence of any commercial or financial relationships that could be construed as a potential conflict of interest.

## Abbreviations

AD: Alzheimer's disease
ROC: receiver operating characteristic
Aβ: amyloid-β
NFT: neurofibrillary tangles
CSF: cerebrospinal fluid
MCI: mild cognition impairment
ELISA: enzyme-linked immunosorbent assay
DEPs: differentially expressed probes
FDR: false discovery rate
FC: fold change
SVM: support vector machines
DSM-IV: Diagnostic and Statistical Manual of Mental Disorders-Fourth Edition
BBB: blood-brain barrier
AUC: area under the curve
ROS: reactive oxygen species
SEM: standard errors of the means.

## References

[1] Blennow K de Leon MJ Zetterberg H. Alzheimer's disease. Lancet (2006) 368:387–403. 10.1016/S0140-6736(06)69113-7 16876668

[2] Inoue K Tsutsui H Akatsu H Hashizume Y Matsukawa N Yamamoto T. Metabolic profiling of Alzheimer's disease brains. Sci Rep. (2013) 3:2364. 10.1038/srep02364 23917584 PMC3734482

[3] Prince M Jackson J World Alzheimer Report 2009. Alzheimer's Disease International (2009).

[4] Daffner KR Current approaches to the clinical diagnosis of Alzheimer's disease. In: Scinto LFM Daffner KR editors. Early Diagnosis of Alzheimer's Disease Totowa, NJ: Humana Press (2000). p. 29–64.

[5] Blennow K Hampel H. CSF markers for incipient Alzheimer's disease. Lancet Neurol. (2003) 2:605–13. 10.1016/S1474-4422(03)00530-1 14505582

[6] Sunderland T Gur RE Arnold SE. The use of biomarkers in the elderly: current and future challenges. Biol Psychiatry (2005) 58:272–6. 10.1016/j.biopsych.2005.05.016 16018985

[7] de Almeida SM Shumaker SD LeBlanc SK Delaney P Marquie-Beck J Ueland S. Incidence of post-dural puncture headache in research volunteers. Headache (2011) 51:1503–10. 10.1111/j.1526-4610.2011.01959.x 21797856 PMC3217171

[8] Ray S Britschgi M Herbert C Takeda-Uchimura Y Boxer A Blennow K. Classification and prediction of clinical Alzheimer's diagnosis based on plasma signaling proteins. Nature Med. (2007) 13:1359–62. 10.1038/nm1653 17934472

[9] Liao PC Yu L Kuo CC Lin CJ Kuo YM. Proteomics analysis of plasma for potential biomarkers in the diagnosis of Alzheimer's disease. Proteomics Clin Appl. (2007) 1:506–12. 10.1002/prca.200600684 21136702

[10] Lovell MA Markesbery WR. Oxidative damage in mild cognitive impairment and early Alzheimer's disease. J Neurosci Res. (2007) 85:3036–40. 10.1002/jnr.21346 17510979

[11] Pratico D Clark CM Lee VM Trojanowski JQ Rokach J FitzGerald GA. Increased 8,12-iso-iPF2alpha-VI in Alzheimer's disease: correlation of a noninvasive index of lipid peroxidation with disease severity. Ann Neurol. (2000) 48:809–12. 10.1002/1531-8249(200011)48:5andlt;809::AID-ANA19andgt;3.0.CO;2-9 11079549

[12] Pratico D Clark CM Liun F Rokach J Lee VY Trojanowski JQ. Increase of brain oxidative stress in mild cognitive impairment: a possible predictor of Alzheimer disease. Arch Neurol. (2002) 59:972–6. 10.1001/archneur.59.6.972 12056933

[13] Bantscheff M Schirle M Sweetman G Rick J Kuster B. Quantitative mass spectrometry in proteomics: a critical review. Anal Bioanal Chem. (2007) 389:1017–31. 10.1007/s00216-007-1486-6 17668192

[14] Edgar R Domrachev M Lash AE. Gene expression omnibus: NCBI gene expression and hybridization array data repository. Nucleic Acids Res. (2002) 30:207–10. 10.1093/nar/30.1.207 11752295 PMC99122

[15] Berchtold NC Cribbs DH Coleman PD Rogers J Head E Kim R. Gene expression changes in the course of normal brain aging are sexually dimorphic. Proc Natl Acad Sci USA. (2008) 105:15605–10. 10.1073/pnas.0806883105 18832152 PMC2563070

[16] Berchtold NC Coleman PD Cribbs DH Rogers J Gillen DL Cotman CW. Synaptic genes are extensively downregulated across multiple brain regions in normal human aging and Alzheimer's disease. Neurobiol Aging (2013) 34:1653–61. 10.1016/j.neurobiolaging.2012.11.024 23273601 PMC4022280

[17] Liang WS Dunckley T Beach TG Grover A Mastroeni D Walker DG. Gene expression profiles in anatomically and functionally distinct regions of the normal aged human brain. Physiol Genomics (2007) 28:311–22. 10.1152/physiolgenomics.00208.2006 17077275 PMC2259385

[18] Irizarry RA Hobbs B Collin F Beazer-Barclay YD Antonellis KJ Scherf U. Exploration, normalization, and summaries of high density oligonucleotide array probe level data. Biostatistics (2003) 4:249–64. 10.1093/biostatistics/4.2.249 12925520

[19] Massey FJ The Kolmogorov-Smirnov test for goodness of fit. J Am Statist Assoc. (1951) 46:68–78. 10.1080/01621459.1951.10500769

[20] Mann HBW Donald R On a test of whether one of two random variables is stochastically larger than the other. Ann Math Statist. (1947) 18:50–60. 10.1214/aoms/1177730491

[21] Benjamini YHY (1995). Controlling the false discovery rate: a practical and powerful approach to multiple testing. J Royal Statist Soc. 57:289–300.

[22] Cui J Liu Q Puett D Xu Y. Computational prediction of human proteins that can be secreted into the bloodstream. Bioinformatics (2008) 24:2370–5. 10.1093/bioinformatics/btn418 18697770 PMC2562011

[23] Souza BF Carvalho AP. Gene selection based on multi-class support vector machines and genetic algorithms. Genet Mol Res. (2005) 4:599–607. 16342045

[24] McDonald JH (Ed.). G–test of goodness-of-fit. In: Handbook of Biological Statistics (4th edn). Baltimore, MD: Sparky House Publishing (2014). p. 53–8.

[25] Fawcett T An introduction to ROC analysis. Pattern Recogni Lett. (2006) 27:861–74. 10.1016/j.patrec.2005.10.010

[26] Robin X Turck N Hainard A Tiberti N Lisacek F Sanchez JC. pROC: an open-source package for R and S+ to analyze and compare ROC curves. BMC Bioinformatics (2011) 12:77. 10.1186/1471-2105-12-77 21414208 PMC3068975

[27] Yao F Hong X Li S Zhang Y Zhao Q Du W. Urine-based biomarkers for alzheimer's disease identified through coupling computational and experimental methods. J Alzheimers Dis. (2018) 65:421–31. 10.3233/JAD-180261 30040720

[28] Ruan Q D'Onofrio G Sancarlo D Greco A Yu Z. Potential fluid biomarkers for pathological brain changes in Alzheimer's disease: implication for the screening of cognitive frailty. Mol Med Rep. (2016) 14:3184–98. 10.3892/mmr.2016.5618 27511317 PMC5042792

[29] Zipser BD Johanson CE Gonzalez L Berzin TM Tavares R Hulette CM. Microvascular injury and blood-brain barrier leakage in Alzheimer's disease. Neurobiol Aging (2007) 28:977–86. 10.1016/j.neurobiolaging.2006.05.016 16782234

[30] Erickson MA Banks WA. Blood-brain barrier dysfunction as a cause and consequence of Alzheimer's disease. J Cereb Blood Flow Metab. (2013) 33:1500–13. 10.1038/jcbfm.2013.135 23921899 PMC3790938

[31] Guntert A Campbell J Saleem M O'Brien DP Thompson AJ Byers HL. Plasma gelsolin is decreased and correlates with rate of decline in Alzheimer's disease. J Alzheimers Dis. (2010) 21:585–96. 10.3233/JAD-2010-100279 20571216

[32] Yu HL. Aberrant profiles of native and oxidized glycoproteins in Alzheimer plasma. Proteomics (2004) 3:2240–8. 10.1002/pmic.200300475 14595822

[33] Ijsselstijn L Dekker LJM Stingl C van der Weiden MM Hofman A Kros JM. Serum levels of pregnancy zone protein are elevated in presymptomatic Alzheimer's disease. J Proteome Res. (2011) 10:4902–10. 10.1021/pr200270z 21879768

[34] Doecke JD Laws SM Faux NG Wilson W Burnham SC Lam CP. Blood-based protein biomarkers for diagnosis of Alzheimer disease. Arch Neurol. (2012) 69:1318–25. 10.1001/archneurol.2012.1282 22801742 PMC6287606

[35] Mhyre TR Loy R Tariot PN Profenno LA Maguire-Zeiss KA Zhang D. Proteomic analysis of peripheral leukocytes in Alzheimer's disease patients treated with divalproex sodium. Neurobiol Aging (2008) 29:1631–43. 10.1016/j.neurobiolaging.2007.04.004 17521776 PMC2621111

[36] Cutler P Akuffo EL Bodnar WM Briggs DM Davis JB Debouck CM. Proteomic identification and early validation of complement 1 inhibitor and pigment epithelium -derived factor: two novel biomarkers of Alzheimer's disease in human plasma. Proteomics Clini Appl. (2008) 2:467–77. 10.1002/prca.200780101 21136851

[37] Wang C Cui Y Yang J Zhang J Yuan D Wei Y. Combining serum and urine biomarkers in the early diagnosis of mild cognitive impairment that evolves into Alzheimer's disease in patients with the apolipoprotein E 4 genotype. Biomarkers (2015) 20:84–8. 10.3109/1354750X.2014.994036 25532446

[38] Kim BY Lee SH Graham PL Angelucci F Lucia A Pareja-Galeano H. Peripheral brain-derived neurotrophic factor levels in Alzheimer's disease and mild cognitive impairment: a comprehensive systematic review and meta-analysis (2016). Mol Neurobiol. 54:7297–311. 10.1007/s12035-016-0192-9 27815832

[39] Huang DW Sherman BT Lempicki RA Bioinformatics enrichment tools: paths toward the comprehensive functional analysis of large gene lists. Nucleic Acids Res. (2009) 37:1–13. 10.1093/nar/gkn923 19033363 PMC2615629

[40] Becker KG Barnes KC Bright TJ Wang SA. The genetic association database. Nat Genet. (2004) 36:431–2. 10.1038/ng0504-431 15118671

[41] Kanehisa M Goto S. KEGG: Kyoto Encyclopedia of Genes and Genomes. Nucleic Acids Res. (2000) 28:27–30. 10.1093/nar/28.1.27 10592173 PMC102409

[42] Rappaport N Twik M Nativ N Stelzer G Bahir I Stein TI. MalaCards: a comprehensive automatically-mined database of human diseases. Curr Protoc Bioinformatics (2014) 47, 1 24 1–19. 10.1002/0471250953.bi0124s47 25199789

[43] Handen A Ganapathiraju MK. LENS: web-based lens for enrichment and network studies of human proteins. BMC Med Genomics (2015) 8 (Suppl. 4):S2. 10.1186/1755-8794-8-S4-S2 26680011 PMC4682415

[44] Padurariu M Ciobica A Lefter R Serban IL Stefanescu C Chirita R. The oxidative stress hypothesis in Alzheimer's disease. Psychiatr Danub. (2013) 25:401–9. 24247053

[45] Zimmer ER Leuzy A Bhat V Gauthier S Rosa-Neto P. *In vivo* tracking of tau pathology using positron emission tomography (PET) molecular imaging in small animals. Transl Neurodegener. (2014) 3:6. 10.1186/2047-9158-3-6 24628994 PMC3995516

[46] Zheng L Roberg K Jerhammar F Marcusson J Terman A. Oxidative stress induces intralysosomal accumulation of Alzheimer amyloid beta-protein in cultured neuroblastoma cells. Ann N Y Acad Sci. (2006) 1067:248–51. 10.1196/annals.1354.032 16803994

[47] Xie AM Gao J Xu L Meng DM. Shared mechanisms of neurodegeneration in Alzheimer's disease and Parkinson's disease. Biomed Res Int. (2014) 2014:648740. 10.1155/2014/648740 24900975 PMC4037122

[48] Peng M Jia JP Qin W. Plasma gelsolin and matrix metalloproteinase 3 as potential biomarkers for Alzheimer disease. Neurosci Lett. (2015) 595:116–21. 10.1016/j.neulet.2015.04.014 25864780

[49] Shen LM Liao LP Chen C Guo Y Song DL Wang Y. Proteomics analysis of blood serums from Alzheimer's disease patients using iTRAQ labeling technology. J Alzheimers Dis. (2017) 56:361–78. 10.3233/JAD-160913 27911324

[50] Ji L Zhao X Hua Z. Potential therapeutic implications of gelsolin in Alzheimer's disease. J Alzheimers Dis. (2015) 44:13–25. 10.3233/JAD-141548 25208622

[51] Yang WZ Chauhan A Mehta S Mehta P Gu F Chauhan V. Trichostatin A increases the levels of plasma gelsolin and amyloid beta-protein in a transgenic mouse model of Alzheimer's disease. Life Sci. (2014) 99:31–6. 10.1016/j.lfs.2014.01.064 24486299

[52] Acheson A Conover JC Fandl JP Dechiara TM Russell M Thadani A. A bdnf autocrine loop in adult sensory neurons prevents cell death. Nature (1995) 374:450–3. 10.1038/374450a0 7700353

[53] Huang EJ Reichardt LF. Neurotrophins: roles in neuronal development and function. Ann Rev Neurosci. (2001) 24:677–736. 10.1146/annurev.neuro.24.1.677 11520916 PMC2758233

[54] Mattson MP. Glutamate and neurotrophic factors in neuronal plasticity and disease. Ann N Y Acad Sci. (2008) 1144:97–112. 10.1196/annals.1418.005 19076369 PMC2614307

[55] Braun DJ Kalinin S Feinstein DL. Conditional depletion of hippocampal brain-derived neurotrophic factor exacerbates neuropathology in a mouse model of Alzheimer's disease. ASN Neuro. (2017) 9:1759091417696161. 10.1177/1759091417696161 28266222 PMC5415058

[56] Hernandez-Guillamon M Delgado P Ortega L Pares M Rosell A Garcia-Bonilla L. Neuronal TIMP-1 release accompanies astrocytic MMP-9 secretion and enhances astrocyte proliferation induced by beta-amyloid 25-35 fragment. J Neurosci Res. (2009) 87:2115–25. 10.1002/jnr.22034 19235898

[57] Mroczko B Koper OM Groblewska M Zboch M Kulczynska-Przybik A Szmitkowski M Matrix metalloproteinase-9 (mmp-9) and its tissue inhibitor-1 (timp-1) as biomarkers of alzheimer's disease. J Alzheimer's Dis. (2014) 10:P520 10.1016/j.jalz.2014.05.811

[58] Wang XX Tan MS Yu JT Tan L. Matrix metalloproteinases and their multiple roles in Alzheimer's disease. Biomed Res Int. (2014) 2014:908636. 10.1155/2014/908636 25050378 PMC4094696

[59] Lorenzl S Albers DS Relkin N Ngyuen T Hilgenberg SL Chirichigno J. Increased plasma levels of matrix metalloproteinase-9 in patients with Alzheimer's disease. Neurochem Int. (2003) 43:191–6. 10.1016/S0197-0186(03)00004-4 12689599

[60] DiBattista AM Dumanis SB Song JM Bu G Weeber E Rebeck GW. Very low density lipoprotein receptor regulates dendritic spine formation in a RasGRF1/CaMKII dependent manner. Biochim Biophys Acta (2015) 1853:904–17. 10.1016/j.bbamcr.2015.01.015 25644714 PMC4580245

[61] Nakamura Y Yamamoto M Kumamaru E. Significance of the variant and full-length forms of the very low density lipoprotein receptor in brain. Brain Res. (2001) 922:209–15. 10.1016/S0006-8993(01)03170-5 11743951

[62] Lane-Donovan C Herz J. The ApoE receptors Vldlr and Apoer2 in central nervous system function and disease. J Lipid Res. (2017) 58:1036–43. 10.1194/jlr.R075507 28292942 PMC5454520

[63] Schilling S Mehr A Ludewig S Stephan J Zimmermann M August A. APLP1 Is a synaptic cell adhesion molecule, supporting maintenance of dendritic spines and basal synaptic transmission. J Neurosci. (2017) 37:5345–65. 10.1523/JNEUROSCI.1875-16.2017 28450540 PMC6596463

[64] Del Prete D Rice RC Rajadhyaksha AM D'Adamio L. Amyloid Precursor Protein (APP) may act as a substrate and a recognition unit for CRL4CRBN and Stub1 E3 ligases facilitating ubiquitination of proteins involved in presynaptic functions and neurodegeneration. J Biol Chem. (2016) 291:17209–27. 10.1074/jbc.M116.733626 27325702 PMC5016122

