# Supplementary material for: Identification of Blood Biomarkers for Alzheimer's Disease Through Computational Prediction and Experimental Validation
Source: Front Neurol. 2019 Jan 8;9:1158. doi: 10.3389/fneur.2018.01158 (PMC6331438; doi:10.3389/fneur.2018.01158)
Supplement: Supplementary file 1 [file Data_Sheet_1.docx]

Supplementary Material

**Identification of Blood B****iomarkers for Alzheimer’s Disease through Computational Prediction and Experimental Validation**

Fang Yao, Kaoyuan Zhang, Yan Zhang, Yi Guo, Aidong Li, Shifeng Xiao, Qiong Liu*, Liming Shen*, Jiazuan Ni

*** Correspondence:** Dr. Qiong Liu: liuqiong@szu.edu.cn, Dr. Liming Shen: slm@szu.edu.cn

# Supplementary Figures

Collection of tissue-based gene expression data of AD

Identification of differentially expressed genes in AD

Prediction of AD-related blood-secretory proteins

Collection of serum samples from AD and healthy individuals

Validation of potential protein biomarkers of AD by ELISA experiments and western blot analyses

Functional analysis on differentially expressed genes

Selection of blood proteins for experimental validation by functional analysis and literature survey

**Supplementary Figure S1.** **The schematic diagram of the workflow in this study.**


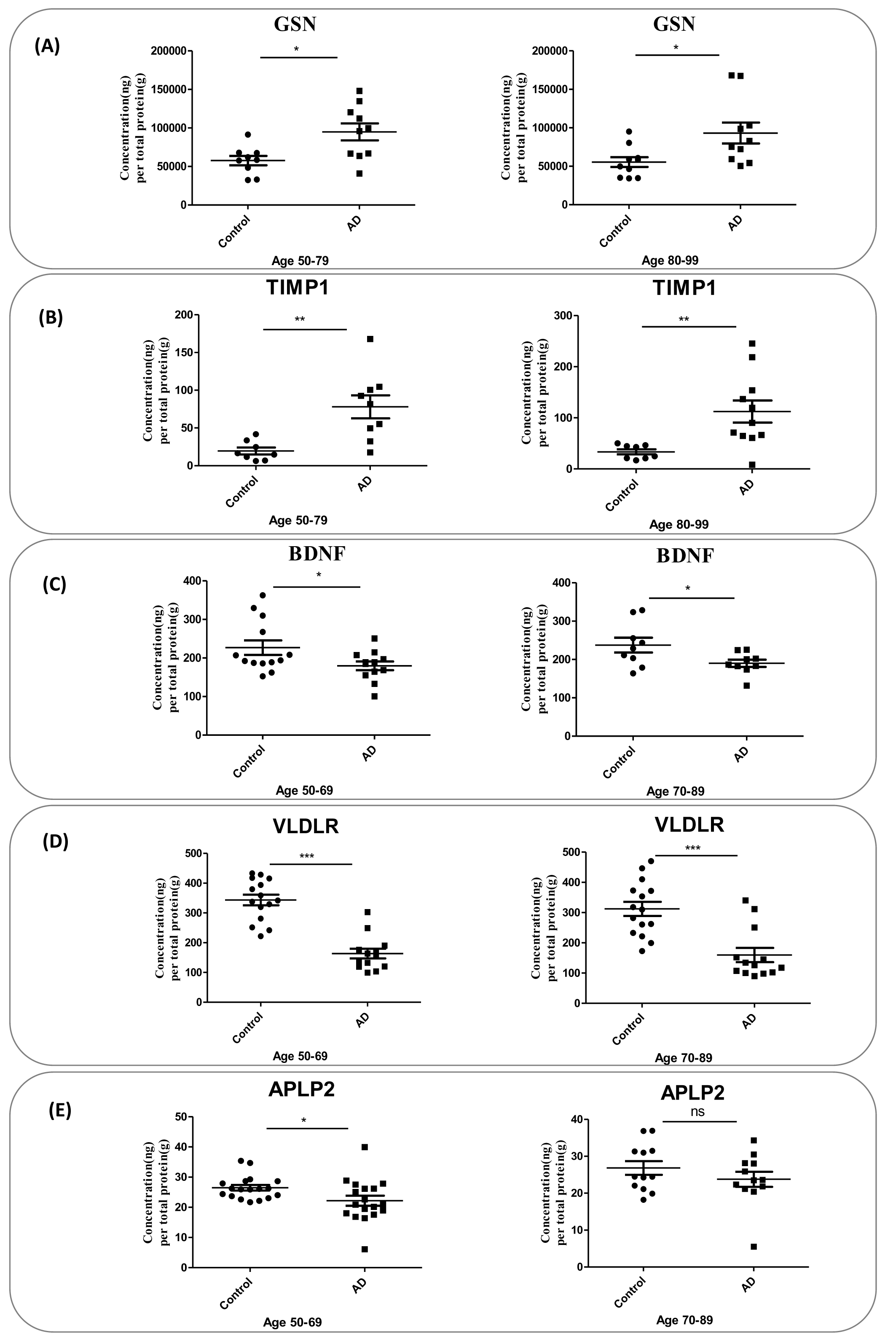


**Supplementary Figure S2.** **The concentrations of five proteins measured using ELISA in AD samples versus controls according to categories of different age stages.** *: p < 0.05; **: p < 0.005; ***: p <0.0005; ns: no significance.


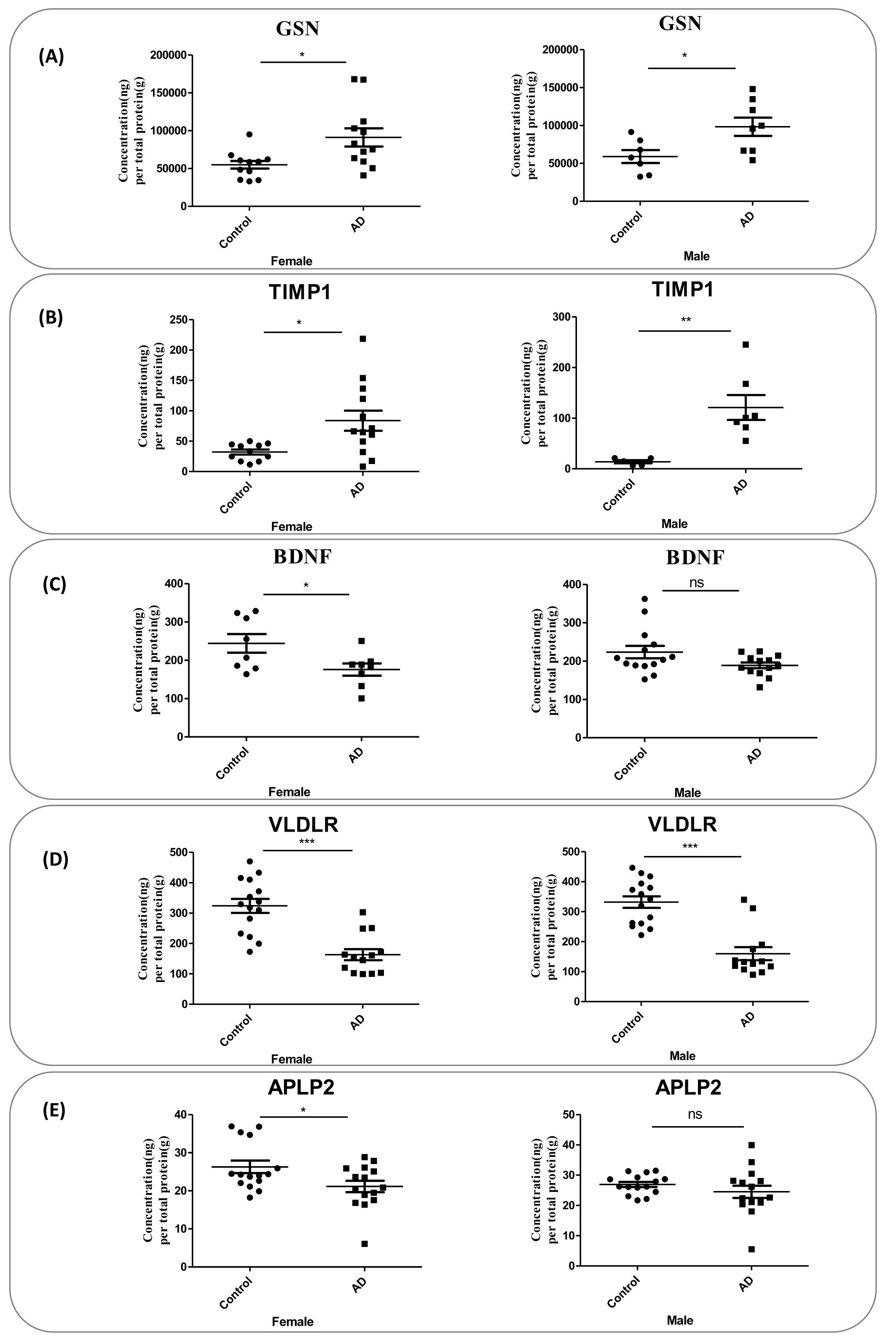


**Supplementary Figure S3.** **The concentrations of five proteins measured using ELISA in AD samples versus controls according to categories of different genders.** *: p < 0.05; **: p < 0.005; ***: p <0.0005; ns: no significance.

# Supplementary Tables

## Supplementary Table S1. The up- and down-regulated genes predicted to encode blood-secretory proteins.

| **# The up-regulated genes predicted to encode secretory-blood proteins** | | | | | | |
| --- | --- | --- | --- | --- | --- | --- |
| **Probe ID** | **Uniprot ID** | **Gene_symbol** | **GSE48350_FDR** | **GSE48350_FC** | **GSE5281_FDR** | **GSE5281_FC** |
| 222784_at | Q9H4F8 | SMOC1 | 0.000538431 | 1.233520743 | 0.002882 | 1.312929 |
| 224215_s_at | O00548 | DLL1 | 3.07E-06 | 1.413549747 | 6.62E-06 | 1.5717 |
| 213994_s_at | Q9HCB6 | SPON1 | 0.04948284 | 1.2010302 | 0.001742 | 1.592642 |
| 227821_at | Q8N135 | LGI4 | 7.28E-06 | 1.231852929 | 0.001574 | 1.294024 |
| 225582_at | Q8IWB1 | ITPRIP | 2.32E-06 | 1.28238583 | 4.62E-12 | 1.75047 |
| 235238_at | Q6S5L8 | SHC4 | 0.001087342 | 1.226986174 | 0.005262 | 1.236289 |
| 229378_at | Q6ZVD7 | STOX1 | 0.001054172 | 1.205605947 | 0.004169 | 1.239626 |
| 220109_at | P02787 | TF | 0.002323768 | 1.383703417 | 4.18E-05 | 1.201536 |
| 214064_at | P02787 | TF | 0.000605498 | 1.343315121 | 0.016273 | 1.201055 |
| 205073_at | P51589 | CYP2J2 | 0.000176257 | 1.261902529 | 0.013455 | 1.339378 |
| 202193_at | P53671 | LIMK2 | 2.99E-05 | 1.328069211 | 4.81E-05 | 1.61853 |
| 202185_at | O60568 | PLOD3 | 1.58E-05 | 1.248784774 | 1.64E-06 | 1.344315 |
| 213905_x_at | P21810 | BGN | 5.37E-06 | 1.242906846 | 3.54E-09 | 2.079118 |
| 228224_at | P51888 | PRELP | 8.34E-05 | 1.297336659 | 2.82E-13 | 1.627494 |
| 204223_at | P51888 | PRELP | 0.000336612 | 1.303132898 | 2.82E-08 | 1.911375 |
| 209395_at | P36222 | CHI3L1 | 6.91E-07 | 1.930580339 | 0.000152 | 2.130503 |
| 209396_s_at | P36222 | CHI3L1 | 7.07E-07 | 1.881030496 | 0.000158 | 2.010138 |
| 218831_s_at | P55899 | FCGRT | 9.48E-06 | 1.258352608 | 0.000112 | 1.26998 |
| 202040_s_at | P29375 | KDM5A | 6.67E-07 | 1.237315027 | 1.19E-06 | 1.394029 |
| 229876_at | P46020 | PHKA1 | 0.002476822 | 1.21988092 | 0.002221 | 1.324846 |
| 229850_at | Q06136 | KDSR | 1.71E-05 | 1.273180642 | 0.005819 | 1.473423 |
| 236361_at | Q8N3T1 | GALNT15 | 8.33E-06 | 1.564901279 | 8.18E-11 | 1.710393 |
| 239461_at | Q8N3T1 | GALNT15 | 1.82E-05 | 1.452221108 | 1.80E-06 | 1.600424 |
| 228501_at | Q8N3T1 | GALNT15 | 1.35E-05 | 1.663100756 | 0.013221 | 1.410479 |
| 221623_at | Q96GW7 | BCAN | 3.02E-05 | 1.234818285 | 0.004432 | 1.291333 |
| 205801_s_at | Q8IV61 | RASGRP3 | 0.013933662 | 1.263233736 | 0.007551 | 1.26539 |
| 220591_s_at | Q5JST6 | EFHC2 | 0.000413213 | 1.271836049 | 0.000989 | 1.40823 |
| 1554479_a_at | Q9Y2G2 | CARD8 | 3.55E-08 | 1.45176855 | 0.000118 | 1.409828 |
| 204984_at | O75487 | GPC4 | 0.00036333 | 1.205909849 | 0.020879 | 1.23329 |
| 209295_at | O14763 | TNFRSF10B | 3.98E-07 | 1.418040581 | 3.22E-09 | 2.257277 |
| 215847_at | Q9BVR0 | HERC2P3 | 0.005957401 | 1.249817628 | 3.51E-07 | 1.435541 |
| 221009_s_at | Q9BY76 | ANGPTL4 | 0.00329529 | 1.242519244 | 0.048985 | 1.313533 |
| 209023_s_at | Q8N3U4 | STAG2 | 4.80E-07 | 1.308269588 | 5.65E-11 | 2.442543 |
| 209022_at | Q8N3U4 | STAG2 | 7.58E-09 | 1.332663352 | 0.043617 | 1.332538 |
| 1007_s_at | Q08345 | DDR1 | 3.08E-05 | 1.336102201 | 1.81E-14 | 2.030483 |
| 208779_x_at | Q08345 | DDR1 | 1.75E-05 | 1.341539564 | 1.76E-10 | 1.941367 |
| 210749_x_at | Q08345 | DDR1 | 1.61E-05 | 1.363444349 | 9.08E-10 | 1.86424 |
| 207169_x_at | Q08345 | DDR1 | 1.79E-05 | 1.368274941 | 1.21E-09 | 1.912491 |
| 212852_s_at | P10155 | TROVE2 | 6.49E-06 | 1.237904728 | 5.03E-09 | 1.730108 |
| 203903_s_at | Q9BQS7 | HEPH | 0.000101317 | 1.328018058 | 3.81E-06 | 1.998508 |
| 210357_s_at | Q9NWM0 | SMOX | 4.40E-07 | 1.303080846 | 8.37E-11 | 1.696862 |
| 204153_s_at | O00587 | MFNG | 2.89E-06 | 1.226132265 | 0.000557 | 1.229948 |
| 211726_s_at | Q99518 | FMO2 | 3.31E-08 | 1.290216426 | 4.42E-07 | 1.358624 |
| 208683_at | P17655 | CAPN2 | 4.28E-10 | 1.39588801 | 0.020432 | 1.352733 |
| 201876_at | Q15165 | PON2 | 1.80E-05 | 1.336270403 | 1.43E-08 | 2.036346 |
| 235529_x_at | Q9Y3Z3 | SAMHD1 | 5.95E-07 | 1.324382771 | 2.54E-14 | 1.925649 |
| 235964_x_at | Q9Y3Z3 | SAMHD1 | 4.49E-06 | 1.271440916 | 5.50E-12 | 1.659087 |
| 1559882_at | Q9Y3Z3 | SAMHD1 | 6.07E-06 | 1.259811136 | 2.02E-06 | 1.271966 |
| 234987_at | Q9Y3Z3 | SAMHD1 | 1.04E-05 | 1.313427728 | 0.00376 | 1.362335 |
| 213578_at | P36894 | BMPR1A | 2.98E-07 | 1.271145364 | 0.000911 | 1.478327 |
| 242918_at | P49321 | NASP | 2.93E-05 | 1.4199039 | 8.20E-07 | 2.286789 |
| 221541_at | Q9H0B8 | CRISPLD2 | 0.004470724 | 1.249445399 | 0.005998 | 1.361663 |
| 225890_at | Q9BQP7 | MGME1 | 5.32E-05 | 1.220266569 | 6.25E-05 | 1.373742 |
| 238886_at | P49755 | TMED10 | 8.71E-06 | 1.271436903 | 1.15E-08 | 1.600369 |
| 209341_s_at | O14920 | IKBKB | 2.24E-08 | 1.315619646 | 2.52E-06 | 1.600921 |
| 209160_at | P42330 | AKR1C3 | 2.33E-07 | 1.509780666 | 0.001627 | 1.503687 |
| 236163_at | Q8N485 | LIX1 | 0.007593805 | 1.27277909 | 0.000571 | 1.213552 |
| 203973_s_at | P49716 | CEBPD | 7.96E-05 | 1.511747282 | 9.38E-11 | 2.061073 |
| 240440_at | Q9BXD5 | NPL | 2.89E-06 | 1.536176601 | 3.74E-07 | 2.038019 |
| 222162_s_at | Q9UHI8 | ADAMTS1 | 0.007358229 | 1.299694983 | 0.004712 | 1.579296 |
| 207643_s_at | P19438 | TNFRSF1A | 8.01E-05 | 1.31039291 | 1.28E-07 | 1.696513 |
| 235155_at | Q9BUT1 | BDH2 | 4.60E-08 | 1.495362858 | 1.59E-06 | 1.645746 |
| 244084_at | Q96NN9 | AIFM3 | 2.54E-05 | 1.241412331 | 0.021879 | 1.25533 |
| 202245_at | P48449 | LSS | 1.21E-07 | 1.363800777 | 0.000169 | 1.34516 |
| 201666_at | P01033 | TIMP1 | 0.001389203 | 1.303668166 | 0.001886 | 1.347768 |
| 231179_at | Q96PC2 | IP6K3 | 2.33E-05 | 1.539421921 | 4.13E-05 | 1.341976 |
| 213110_s_at | P29400 | COL4A5 | 0.026151199 | 1.320058085 | 0.000488 | 1.388106 |
| 224569_s_at | Q7Z5L9 | IRF2BP2 | 2.67E-06 | 1.307250706 | 5.25E-08 | 2.168748 |
| 224570_s_at | Q7Z5L9 | IRF2BP2 | 7.96E-05 | 1.204598673 | 0.004856 | 1.31423 |
| 209791_at | Q9Y2J8 | PADI2 | 0.000841551 | 1.229106698 | 3.96E-06 | 1.487506 |
| 209933_s_at | Q9UGN4 | CD300A | 0.000334376 | 1.223240004 | 0.000989 | 1.251328 |
| 213503_x_at | P07355 | ANXA2 | 5.29E-05 | 1.392097659 | 8.76E-05 | 1.468211 |
| 210427_x_at | P07355 | ANXA2 | 6.95E-05 | 1.408497215 | 0.00029 | 1.466784 |
| 201590_x_at | P07355 | ANXA2 | 5.98E-05 | 1.401893995 | 0.004525 | 1.375159 |
| 209258_s_at | Q9UQE7 | SMC3 | 0.00021162 | 1.220233137 | 3.40E-12 | 3.08558 |
| 223592_s_at | Q8IUD6 | RNF135 | 4.96E-07 | 1.470159468 | 4.00E-07 | 1.920889 |
| 201042_at | P21980 | TGM2 | 0.001666063 | 1.263991324 | 0.000321 | 1.408335 |
| 204158_s_at | Q13488 | TCIRG1 | 4.84E-05 | 1.210211897 | 3.09E-08 | 1.540095 |
| 208795_s_at | P33993 | MCM7 | 6.96E-07 | 1.279000337 | 5.54E-08 | 1.477086 |
| 228121_at | P61812 | TGFB2 | 4.14E-08 | 1.468200104 | 1.29E-10 | 2.003134 |
| 210051_at | O95398 | RAPGEF3 | 4.94E-06 | 1.321764656 | 1.09E-07 | 1.751141 |
| 238025_at | Q8NB16 | MLKL | 2.72E-05 | 1.278032887 | 2.58E-05 | 1.297837 |
| 1558487_a_at | Q7Z7H5 | TMED4 | 0.000127299 | 1.216087849 | 7.86E-06 | 1.431256 |
| 233813_at | Q96T49 | PPP1R16B | 0.0001006 | 1.227303705 | 5.43E-08 | 2.003735 |
| 230214_at | Q9Y6F6 | MRVI1 | 0.005161109 | 1.366248461 | 4.64E-07 | 1.941808 |
| 226047_at | Q9Y6F6 | MRVI1 | 0.0010974 | 1.374936296 | 8.23E-06 | 1.872817 |
| 208659_at | O00299 | CLIC1 | 6.00E-06 | 1.370897814 | 1.49E-05 | 1.459119 |
| 242579_at | O00238 | BMPR1B | 1.58E-06 | 1.529320022 | 4.63E-11 | 2.336563 |
| 229975_at | O00238 | BMPR1B | 9.07E-05 | 1.450748694 | 1.10E-05 | 1.761567 |
| 201589_at | Q14683 | SMC1A | 2.97E-05 | 1.264118348 | 2.25E-05 | 1.669441 |
| 234801_s_at | Q9NUB1 | ACSS1 | 7.12E-07 | 1.290701965 | 3.93E-06 | 1.372589 |
| 206101_at | O94769 | ECM2 | 0.000186576 | 1.569580702 | 2.05E-05 | 1.445075 |
| 215248_at | Q13322 | GRB10 | 4.13E-06 | 1.260655817 | 0.002372 | 1.332313 |
| 209747_at | P10600 | TGFB3 | 1.60E-05 | 1.276380883 | 0.002346 | 1.276344 |
| 202625_at | P07948 | LYN | 0.000379665 | 1.201783283 | 0.000421 | 1.233736 |
| 1555889_a_at | O75718 | CRTAP | 4.26E-10 | 1.286483065 | 3.69E-06 | 1.535669 |
| 226656_at | O75718 | CRTAP | 5.19E-09 | 1.300196925 | 0.000234 | 1.403177 |
| 202023_at | P20827 | EFNA1 | 0.000387241 | 1.288353204 | 7.55E-07 | 1.590316 |
| 201289_at | O00622 | CYR61 | 0.000873338 | 1.398188422 | 0.009746 | 1.342606 |
| 217456_x_at | P13747 | HLA-E | 3.34E-06 | 1.248036803 | 5.40E-10 | 1.715042 |
| 200905_x_at | P13747 | HLA-E | 1.03E-05 | 1.315117659 | 5.76E-10 | 2.023435 |
| 200904_at | P13747 | HLA-E | 0.000252516 | 1.255572396 | 2.63E-05 | 1.519581 |
| 232424_at | Q9HAZ2 | PRDM16 | 8.16E-05 | 1.338112526 | 8.11E-06 | 2.109218 |
| 1554442_at | O76090 | BEST1 | 4.92E-07 | 1.271565544 | 0.003797 | 1.218368 |
| 1552309_a_at | Q0ZGT2 | NEXN | 1.06E-07 | 1.320986276 | 8.30E-06 | 1.340263 |
| 200677_at | P53801 | PTTG1IP | 2.68E-05 | 1.355135262 | 0.000185 | 1.45693 |
| 203068_at | Q9UJP4 | KLHL21 | 7.58E-08 | 1.229571792 | 4.59E-09 | 1.426706 |
| 234985_at | Q86YD5 | LDLRAD3 | 3.71E-06 | 1.32563153 | 4.06E-06 | 1.676764 |
| 228450_at | Q6IQ23 | PLEKHA7 | 4.28E-06 | 1.362749419 | 0.00028 | 1.230169 |
| 202464_s_at | Q16875 | PFKFB3 | 8.11E-07 | 1.272743635 | 5.19E-10 | 1.890401 |
| 236561_at | P36897 | TGFBR1 | 7.93E-06 | 1.361317294 | 0.007961 | 1.300726 |
| 213553_x_at | P02654 | APOC1 | 1.01E-05 | 1.212929436 | 1.44E-06 | 1.424592 |
| 227401_at | Q8TAD2 | IL17D | 0.000210211 | 1.247114538 | 0.004619 | 1.351183 |
| 228670_at | P60484 | TEP1 | 1.62E-06 | 1.319386309 | 2.43E-06 | 1.346074 |
| 219426_at | Q9H9G7 | AGO3 | 1.20E-05 | 1.323910696 | 6.93E-05 | 1.351519 |
| 203332_s_at | Q92835 | INPP5D | 2.98E-07 | 1.30834756 | 3.74E-09 | 1.546367 |
| 232617_at | P25774 | CTSS | 1.55E-09 | 1.481138797 | 0.00195 | 1.267413 |
| 229344_x_at | Q9ULI2 | RIMKLB | 6.87E-07 | 1.225771918 | 5.25E-08 | 1.40942 |
| 226164_x_at | Q9ULI2 | RIMKLB | 0.000347779 | 1.206232534 | 0.000123 | 1.271375 |
| 205258_at | P09529 | INHBB | 8.58E-05 | 1.227746351 | 3.01E-09 | 1.660658 |
| 225655_at | Q96T88 | UHRF1 | 1.44E-07 | 1.519385635 | 6.16E-10 | 1.521629 |
| 215269_at | P48553 | TRAPPC10 | 0.000135967 | 1.264981392 | 5.25E-08 | 1.574066 |
| 209684_at | Q8WYP3 | RIN2 | 5.09E-07 | 1.430477411 | 5.05E-07 | 2.121042 |
| 227539_at | Q14344 | GNA13 | 6.16E-10 | 1.691247196 | 1.75E-08 | 1.880942 |
| 224761_at | Q14344 | GNA13 | 4.66E-09 | 1.469093138 | 0.038516 | 1.29395 |
| 208991_at | P40763 | STAT3 | 2.95E-06 | 1.251887696 | 6.65E-05 | 1.659207 |
| 211105_s_at | O95644 | NFATC1 | 7.07E-06 | 1.255695506 | 8.11E-06 | 1.450016 |
| 207030_s_at | Q16527 | CSRP2 | 3.51E-05 | 1.361890714 | 0.008941 | 1.44264 |
| 214829_at | Q9UDR5 | AASS | 0.001886737 | 1.239036629 | 1.29E-09 | 1.436565 |
| 227627_at | Q96BR1 | SGK3 | 0.000117161 | 1.261542028 | 7.64E-05 | 1.400002 |
| 200696_s_at | P06396 | GSN | 0.000455291 | 1.291409133 | 0.000216 | 1.596025 |
| 221829_s_at | Q92973 | TNPO1 | 4.72E-08 | 1.298853156 | 5.41E-16 | 2.419073 |
| 209225_x_at | Q92973 | TNPO1 | 1.52E-07 | 1.270838823 | 2.51E-14 | 2.150402 |
| 228956_at | Q16880 | UGT8 | 0.04948284 | 1.277225704 | 0.010607 | 1.484067 |
| 236291_at | Q92781 | RDH5 | 9.03E-06 | 1.227559544 | 1.91E-08 | 1.429054 |
| 213293_s_at | Q8IYM9 | TRIM22 | 1.57E-09 | 1.671750693 | 8.90E-07 | 1.483454 |
| 209369_at | P12429 | ANXA3 | 6.74E-05 | 1.378110592 | 0.004386 | 1.456987 |
| 228028_at | Q75VX8 | GAREML | 1.34E-07 | 1.263924878 | 4.62E-08 | 1.741871 |
| 232015_at | Q75VX8 | GAREML | 4.68E-06 | 1.316503032 | 0.000128 | 1.278834 |

| **The down-regulated genes predicted to encode secretory-blood proteins.** | | | | | | |
| --- | --- | --- | --- | --- | --- | --- |
| **Probe ID** | **Uniprot ID** | **Gene_symbol** | **GSE48350_FDR** | **GSE48350_FC** | **GSE5281_FDR** | **GSE5281_FC** |
| 203110_at | Q14289 | PTK2B | 0.001356 | 0.831029 | 0.001357 | 0.799835 |
| 202852_s_at | Q6PD74 | AAGAB | 8.23E-07 | 0.813539 | 1.15E-06 | 0.593752 |
| 219005_at | Q9UK28 | TMEM59L | 2.51E-09 | 0.722295 | 6.78E-09 | 0.692064 |
| 212958_x_at | P19021 | PAM | 6.28E-06 | 0.82844 | 1.75E-08 | 0.576538 |
| 205721_at | O00451 | GFRA2 | 0.000346 | 0.813574 | 7.13E-06 | 0.714645 |
| 229770_at | Q96MS3 | GLT1D1 | 4.64E-06 | 0.745319 | 3.79E-11 | 0.623664 |
| 212271_at | P28482 | MAPK1 | 2.32E-05 | 0.784985 | 2.39E-08 | 0.458799 |
| 200720_s_at | P61163 | ACTR1A | 1.26E-06 | 0.764828 | 4.48E-13 | 0.407525 |
| 209159_s_at | Q9ULP0 | NDRG4 | 1.68E-06 | 0.734345 | 5.45E-11 | 0.427932 |
| 208693_s_at | P41250 | GARS | 6.33E-06 | 0.802709 | 1.65E-09 | 0.520802 |
| 209080_x_at | O76003 | GLRX3 | 6.34E-07 | 0.83033 | 2.21E-06 | 0.661771 |
| 223651_x_at | Q9UJX2 | CDC23 | 1.95E-06 | 0.800058 | 0.000215 | 0.658488 |
| 227702_at | Q8N118 | CYP4X1 | 4.77E-05 | 0.753729 | 1.55E-06 | 0.571706 |
| 213479_at | P47972 | NPTX2 | 8.82E-06 | 0.563841 | 6.78E-09 | 0.378469 |
| 210124_x_at | O95754 | SEMA4F | 1.86E-06 | 0.827204 | 2.98E-10 | 0.606411 |
| 228660_x_at | O95754 | SEMA4F | 5.38E-07 | 0.812554 | 3.64E-09 | 0.636804 |
| 233150_at | Q7Z4T8 | GALNTL5 | 0.003598 | 0.8033 | 0.00142 | 0.734388 |
| 212461_at | O14977 | AZIN1 | 3.43E-07 | 0.784438 | 7.40E-06 | 0.528547 |
| 229247_at | Q53RD9 | FBLN7 | 4.18E-05 | 0.738212 | 0.000421 | 0.720428 |
| 228503_at | Q9UK32 | RPS6KA6 | 7.80E-07 | 0.799949 | 6.12E-05 | 0.718867 |
| 208838_at | Q86VP6 | CAND1 | 2.87E-06 | 0.815174 | 0.001557 | 0.720915 |
| 205768_s_at | O14975 | SLC27A2 | 3.43E-06 | 0.826606 | 0.000503 | 0.735432 |
| 1556619_at | B4DS77 | SHISA9 | 1.44E-07 | 0.730242 | 0.014759 | 0.792431 |
| 239367_at | P23560 | BDNF | 3.24E-10 | 0.547606 | 5.83E-07 | 0.530404 |
| 206382_s_at | P23560 | BDNF | 4.28E-10 | 0.464378 | 1.42E-05 | 0.545467 |
| 203785_s_at | Q9NUL7 | DDX28 | 6.19E-06 | 0.833121 | 2.22E-08 | 0.690309 |
| 204117_at | P48147 | PREP | 0.000369 | 0.828295 | 7.38E-11 | 0.49421 |
| 37950_at | P48147 | PREP | 0.000217 | 0.810546 | 1.66E-07 | 0.567638 |
| 202283_at | P36955 | SERPINF1 | 1.51E-06 | 0.660057 | 4.07E-08 | 0.4582 |
| 204813_at | P53779 | MAPK10 | 0.000273 | 0.830005 | 1.60E-08 | 0.574876 |
| 219013_at | Q8NCW6 | GALNT11 | 1.34E-08 | 0.798302 | 9.50E-05 | 0.776838 |
| 211630_s_at | P48637 | GSS | 7.67E-11 | 0.814621 | 9.50E-12 | 0.657215 |
| 201415_at | P48637 | GSS | 7.58E-09 | 0.828385 | 3.02E-11 | 0.521488 |
| 203376_at | O60508 | CDC40 | 5.95E-06 | 0.832057 | 1.08E-09 | 0.482688 |
| 203377_s_at | O60508 | CDC40 | 2.66E-05 | 0.775889 | 0.004209 | 0.798596 |
| 205774_at | P00748 | F12 | 2.98E-06 | 0.782878 | 2.26E-06 | 0.669448 |
| 227944_at | P26045 | PTPN3 | 2.85E-09 | 0.670908 | 1.85E-14 | 0.559879 |
| 203997_at | P26045 | PTPN3 | 1.61E-08 | 0.611135 | 3.41E-11 | 0.565478 |
| 208002_s_at | O00154 | ACOT7 | 1.15E-07 | 0.647973 | 1.20E-11 | 0.368712 |
| 215728_s_at | O00154 | ACOT7 | 2.11E-07 | 0.62394 | 8.09E-11 | 0.663308 |
| 222614_at | P57060 | RWDD2B | 0.001006 | 0.814941 | 2.96E-11 | 0.57876 |
| 200695_at | P30153 | PPP2R1A | 2.37E-08 | 0.805419 | 1.51E-08 | 0.520112 |
| 232010_at | Q8N475 | FSTL5 | 2.05E-05 | 0.733854 | 0.029986 | 0.711459 |
| 206013_s_at | O94805 | ACTL6B | 3.96E-06 | 0.644606 | 8.66E-11 | 0.475398 |
| 206014_at | O94805 | ACTL6B | 3.83E-05 | 0.808354 | 5.14E-07 | 0.573305 |
| 217897_at | Q9H0Q3 | FXYD6 | 3.85E-06 | 0.766324 | 5.16E-05 | 0.539242 |
| 203917_at | P78310 | CXADR | 2.11E-06 | 0.801633 | 0.009746 | 0.797445 |
| 233002_at | Q6NUP7 | PPP4R4 | 1.04E-06 | 0.61659 | 0.006456 | 0.656982 |
| 216307_at | Q9Y6T7 | DGKB | 7.56E-07 | 0.613187 | 2.22E-07 | 0.523757 |
| 203762_s_at | Q8TCX1 | DYNC2LI1 | 5.40E-05 | 0.820695 | 2.72E-08 | 0.504659 |
| 211615_s_at | P42704 | LRPPRC | 8.16E-07 | 0.815868 | 1.26E-10 | 0.486912 |
| 211971_s_at | P42704 | LRPPRC | 4.18E-07 | 0.811272 | 2.71E-05 | 0.649637 |
| 236734_at | Q96PX8 | SLITRK1 | 8.27E-06 | 0.80611 | 0.000201 | 0.680428 |
| 222572_at | Q9P0J1 | PDP1 | 0.016897 | 0.799008 | 0.002493 | 0.618241 |
| 227015_at | Q6ICH7 | ASPHD2 | 1.33E-05 | 0.765439 | 2.38E-06 | 0.55866 |
| 222830_at | Q9NZI5 | GRHL1 | 3.30E-06 | 0.816492 | 2.75E-05 | 0.63525 |
| 219945_at | Q9UHL0 | DDX25 | 5.99E-07 | 0.79116 | 1.15E-06 | 0.608163 |
| 202780_at | P55809 | OXCT1 | 3.07E-07 | 0.75688 | 1.69E-08 | 0.446412 |
| 209541_at | P05019 | IGF1 | 2.28E-08 | 0.63106 | 1.26E-05 | 0.659109 |
| 211404_s_at | Q06481 | APLP2 | 0.000302 | 0.832486 | 0.000107 | 0.738963 |
| 202930_s_at | Q9P2R7 | SUCLA2 | 3.17E-05 | 0.782598 | 2.69E-06 | 0.446497 |
| 201839_s_at | P16422 | EPCAM | 6.56E-05 | 0.738017 | 0.011101 | 0.720953 |
| 225689_at | Q8NAT1 | GTDC2 | 5.55E-06 | 0.831017 | 0.015429 | 0.812509 |
| 205346_at | Q16842 | ST3GAL2 | 1.00E-05 | 0.831773 | 4.59E-09 | 0.675467 |
| 230112_at | Q9P2E8 | MARCH4 | 3.16E-09 | 0.692338 | 4.62E-08 | 0.691375 |
| 201714_at | P23258 | TUBG1 | 6.01E-07 | 0.771434 | 8.12E-09 | 0.49881 |
| 226517_at | P54687 | BCAT1 | 0.027739 | 0.828373 | 9.04E-07 | 0.550492 |
| 225285_at | P54687 | BCAT1 | 0.007438 | 0.81118 | 4.06E-06 | 0.561836 |
| 205325_at | Q92561 | PHYHIP | 3.19E-06 | 0.771275 | 9.18E-07 | 0.678096 |
| 227784_s_at | Q8WTW3 | COG1 | 3.09E-06 | 0.812778 | 8.94E-12 | 0.489195 |
| 203517_at | O75431 | MTX2 | 1.92E-06 | 0.7851 | 2.27E-11 | 0.344471 |
| 214321_at | P48745 | NOV | 3.59E-05 | 0.718976 | 0.025049 | 0.776271 |
| 207957_s_at | P05771 | PRKCB | 0.00036 | 0.702376 | 1.07E-06 | 0.441542 |
| 227817_at | P05771 | PRKCB | 3.98E-05 | 0.755479 | 0.017161 | 0.825176 |
| 200613_at | Q96CW1 | AP2M1 | 6.60E-08 | 0.769601 | 1.16E-10 | 0.362585 |
| 203094_at | Q15013 | MAD2L1BP | 6.30E-07 | 0.825126 | 3.23E-11 | 0.568918 |
| 210448_s_at | Q93086 | P2RX5 | 1.44E-07 | 0.769133 | 1.75E-08 | 0.578082 |
| 224994_at | Q13557 | CAMK2D | 1.04E-05 | 0.743587 | 1.44E-07 | 0.511877 |
| 225019_at | Q13557 | CAMK2D | 0.000129 | 0.816419 | 1.78E-05 | 0.631114 |
| 227425_at | Q8NFH8 | REPS2 | 8.64E-05 | 0.760179 | 1.06E-09 | 0.377741 |
| 238681_at | Q8N9F7 | GDPD1 | 0.000435 | 0.795699 | 0.002913 | 0.651621 |
| 204684_at | Q15818 | NPTX1 | 1.05E-06 | 0.713203 | 9.02E-06 | 0.590901 |
| 213646_x_at | P68363 | TUBA1B | 3.00E-07 | 0.830538 | 5.38E-12 | 0.389571 |
| 201090_x_at | P68363 | TUBA1B | 1.48E-07 | 0.820346 | 9.89E-12 | 0.40458 |
| 202432_at | P16298 | PPP3CB | 1.98E-06 | 0.747159 | 3.62E-10 | 0.495539 |
| 223035_s_at | Q9NSD9 | FARSB | 1.40E-08 | 0.806252 | 1.05E-14 | 0.498375 |
| 202260_s_at | P61764 | STXBP1 | 3.51E-05 | 0.772206 | 1.75E-09 | 0.529248 |
| 225781_at | P45984 | MAPK9 | 1.32E-05 | 0.794753 | 8.45E-09 | 0.543482 |
| 210570_x_at | P45984 | MAPK9 | 0.000118 | 0.735672 | 3.63E-07 | 0.717627 |
| 203218_at | P45984 | MAPK9 | 0.000315 | 0.775408 | 2.49E-05 | 0.56484 |
| 218786_at | Q86UY8 | NT5DC3 | 2.39E-05 | 0.79997 | 2.54E-07 | 0.554596 |
| 228763_at | P54098 | MDP1 | 8.82E-05 | 0.824706 | 3.57E-10 | 0.486854 |
| 1554012_at | Q6UXX9 | RSPO2 | 0.047473 | 0.784334 | 5.62E-05 | 0.549133 |
| 230204_at | P10915 | HAPLN1 | 2.52E-07 | 0.743953 | 0.000669 | 0.813236 |
| 205523_at | P10915 | HAPLN1 | 1.71E-06 | 0.783001 | 0.006584 | 0.814403 |
| 202722_s_at | Q06210 | GFPT1 | 3.25E-06 | 0.779123 | 1.30E-05 | 0.660818 |
| 202030_at | O14874 | BCKDK | 4.71E-09 | 0.813103 | 5.59E-06 | 0.724158 |
| 212358_at | Q96DZ5 | CLIP3 | 2.81E-09 | 0.80028 | 3.02E-11 | 0.525493 |
| 228010_at | Q9Y2T4 | PPP2R2C | 2.89E-06 | 0.808391 | 6.28E-07 | 0.742422 |
| 216323_x_at | Q13748 | TUBA3D | 6.81E-07 | 0.820471 | 1.76E-10 | 0.506441 |
| 205899_at | P78396 | CCNA1 | 3.82E-06 | 0.746026 | 1.98E-10 | 0.529103 |
| 235253_at | O60671 | RAD1 | 1.24E-06 | 0.807798 | 0.001645 | 0.793512 |
| 227606_s_at | Q96FJ0 | STAMBPL1 | 0.002026 | 0.816541 | 1.21E-06 | 0.560454 |
| 202949_s_at | Q14192 | FHL2 | 2.44E-07 | 0.666066 | 1.18E-11 | 0.465387 |
| 212242_at | P68366 | TUBA4A | 6.67E-07 | 0.671948 | 5.38E-12 | 0.367285 |
| 205633_s_at | P13196 | ALAS1 | 1.20E-05 | 0.807201 | 5.73E-07 | 0.468983 |
| 1563111_a_at | Q8TBF5 | PIGX | 0.000405 | 0.832134 | 2.05E-06 | 0.832936 |
| 219825_at | Q9NR63 | CYP26B1 | 2.96E-08 | 0.590652 | 0.00085 | 0.690941 |
| 202348_s_at | O14656 | TOR1A | 5.76E-08 | 0.808095 | 1.89E-09 | 0.661363 |
| 209822_s_at | P98155 | VLDLR | 2.91E-07 | 0.754129 | 8.91E-08 | 0.53769 |
| 206213_at | O00744 | WNT10B | 3.24E-09 | 0.779242 | 0.001141 | 0.791082 |
| 205550_s_at | Q9NXR7 | BRE | 1.92E-08 | 0.821754 | 9.83E-14 | 0.548777 |
| 228987_at | Q9NUQ9 | FAM49B | 0.002037 | 0.778022 | 5.78E-07 | 0.459445 |
| 205327_s_at | P27037 | ACVR2A | 6.77E-08 | 0.832951 | 2.03E-05 | 0.788454 |
| 209026_x_at | P07437 | TUBB | 1.18E-07 | 0.747421 | 3.57E-13 | 0.330354 |
| 211714_x_at | P07437 | TUBB | 1.71E-07 | 0.762195 | 9.91E-13 | 0.351587 |
| 226132_s_at | Q5VSG8 | MANEAL | 1.02E-09 | 0.751619 | 1.24E-08 | 0.550981 |
| 222230_s_at | Q9NZ32 | ACTR10 | 9.16E-09 | 0.743447 | 1.88E-08 | 0.480565 |
| 204552_at | Q96PE3 | INPP4A | 7.78E-06 | 0.745788 | 5.06E-10 | 0.499371 |
| 227087_at | Q96PE3 | INPP4A | 7.95E-06 | 0.808445 | 0.001267 | 0.7292 |
| 203340_s_at | O75746 | SLC25A12 | 0.005481 | 0.790803 | 3.51E-08 | 0.495395 |
| 208977_x_at | P68371 | TUBB4B | 1.25E-08 | 0.750444 | 3.42E-14 | 0.341232 |
| 213726_x_at | P68371 | TUBB4B | 1.70E-08 | 0.775929 | 3.89E-13 | 0.408866 |
| 223542_at | Q9BQI6 | ANKRD32 | 0.003429 | 0.81037 | 1.76E-05 | 0.527413 |
| 211855_s_at | O95258 | SLC25A14 | 1.46E-06 | 0.771074 | 2.28E-10 | 0.651995 |
| 204587_at | O95258 | SLC25A14 | 2.91E-07 | 0.83185 | 2.28E-09 | 0.59983 |
| 203594_at | O00442 | RTCA | 2.81E-07 | 0.734972 | 9.76E-05 | 0.584332 |
| 201341_at | O14682 | ENC1 | 4.80E-07 | 0.678685 | 3.42E-10 | 0.320945 |
| 201340_s_at | O14682 | ENC1 | 6.24E-05 | 0.632059 | 0.000944 | 0.614087 |
| 235882_at | Q5VIR6 | VPS53 | 3.43E-06 | 0.784116 | 0.001141 | 0.812099 |
| 215518_at | Q9Y2K9 | STXBP5L | 1.76E-05 | 0.746488 | 7.99E-06 | 0.606904 |
| 219633_at | Q9BTX7 | TTPAL | 5.55E-08 | 0.772817 | 3.52E-05 | 0.826878 |
| 226794_at | Q5T5C0 | STXBP5 | 1.93E-05 | 0.737208 | 0.01599 | 0.813895 |
| 236738_at | F5H4A9 | C3orf80 | 1.76E-05 | 0.622813 | 0.007267 | 0.606577 |
| 209394_at | O95671 | ASMTL | 1.32E-05 | 0.797155 | 2.16E-11 | 0.572427 |
| 36554_at | O95671 | ASMTL | 1.40E-05 | 0.762912 | 1.12E-09 | 0.693704 |
| 36553_at | O95671 | ASMTL | 0.000182 | 0.818226 | 2.57E-08 | 0.584324 |
| 238815_at | Q86UE6 | LRRTM1 | 1.91E-08 | 0.679788 | 1.27E-08 | 0.518379 |
| 215535_s_at | Q99943 | AGPAT1 | 1.89E-12 | 0.831725 | 2.66E-09 | 0.664631 |
| 32836_at | Q99943 | AGPAT1 | 1.29E-11 | 0.82924 | 7.30E-07 | 0.776654 |
| 204967_at | Q13796 | SHROOM2 | 0.001074 | 0.816033 | 2.56E-05 | 0.654751 |
| 227585_at | Q8NBU5 | ATAD1 | 5.32E-06 | 0.791979 | 8.62E-10 | 0.478437 |
| 200786_at | Q99436 | PSMB7 | 1.77E-08 | 0.814356 | 6.49E-12 | 0.385921 |
| 233547_x_at | P54750 | PDE1A | 3.76E-07 | 0.590481 | 3.22E-09 | 0.6471 |
| 242789_at | P54750 | PDE1A | 7.72E-06 | 0.616232 | 9.18E-07 | 0.817532 |
| 236234_at | P54750 | PDE1A | 1.22E-06 | 0.729439 | 9.74E-06 | 0.459803 |
| 208396_s_at | P54750 | PDE1A | 1.46E-05 | 0.736667 | 5.62E-05 | 0.525687 |
| 1558680_s_at | P54750 | PDE1A | 4.03E-06 | 0.609933 | 0.041806 | 0.818702 |
| 222132_s_at | Q53H12 | AGK | 7.91E-07 | 0.810627 | 6.13E-12 | 0.576627 |
| 204794_at | Q05923 | DUSP2 | 7.91E-09 | 0.744238 | 6.89E-05 | 0.781432 |
| 1554680_s_at | Q9ULS6 | KCNS2 | 0.000236 | 0.827295 | 3.23E-06 | 0.768168 |
| 232401_at | Q9ULS6 | KCNS2 | 0.000272 | 0.746755 | 6.22E-06 | 0.56844 |
| 205047_s_at | P08243 | ASNS | 3.13E-06 | 0.772891 | 5.18E-11 | 0.390172 |
| 205632_s_at | O14986 | PIP5K1B | 0.004254 | 0.750863 | 0.000129 | 0.523039 |
| 213849_s_at | Q00005 | PPP2R2B | 3.68E-08 | 0.818922 | 5.45E-11 | 0.54527 |
| 231102_at | Q9UKG9 | CROT | 5.18E-08 | 0.789183 | 5.80E-08 | 0.659646 |
| 1553976_a_at | Q9BVM2 | DPCD | 1.95E-06 | 0.831121 | 1.65E-09 | 0.440458 |
| 225779_at | Q6P1M0 | SLC27A4 | 8.50E-09 | 0.81386 | 1.97E-09 | 0.685892 |
| 1556627_at | Q13474 | DRP2 | 0.000617 | 0.786917 | 7.49E-07 | 0.608528 |
| 203157_s_at | O94925 | GLS | 0.000276 | 0.677482 | 1.41E-10 | 0.381959 |
| 203159_at | O94925 | GLS | 0.010655 | 0.791328 | 1.94E-10 | 0.421267 |
| 203158_s_at | O94925 | GLS | 0.000124 | 0.626725 | 2.66E-10 | 0.547127 |
| 212956_at | Q6ZT07 | TBC1D9 | 9.17E-07 | 0.714386 | 1.43E-08 | 0.375871 |
| 1555910_at | Q8WV60 | PTCD2 | 2.18E-06 | 0.799975 | 1.97E-05 | 0.571883 |
| 219140_s_at | P02753 | RBP4 | 8.27E-09 | 0.57638 | 2.00E-07 | 0.391053 |
| 230306_at | Q4G0F5 | VPS26B | 1.52E-06 | 0.833141 | 1.02E-08 | 0.694547 |
| 202697_at | O43809 | NUDT21 | 8.01E-05 | 0.756087 | 8.84E-07 | 0.772537 |
| 222703_s_at | Q86U90 | YRDC | 1.69E-07 | 0.81756 | 2.16E-06 | 0.79896 |
| 219670_at | Q7L4P6 | BEND5 | 3.00E-06 | 0.751311 | 1.86E-13 | 0.516831 |
| 223708_at | Q9BXJ3 | C1QTNF4 | 2.26E-08 | 0.596812 | 3.68E-10 | 0.453751 |
| 226046_at | P45983 | MAPK8 | 1.75E-06 | 0.736189 | 0.000167 | 0.76707 |
| 210454_s_at | P48051 | KCNJ6 | 0.000502 | 0.773753 | 3.03E-08 | 0.48412 |
| 204186_s_at | Q08752 | PPID | 0.000715 | 0.806728 | 2.38E-05 | 0.583609 |
| 227179_at | Q9NUL3 | STAU2 | 1.89E-07 | 0.739118 | 2.56E-10 | 0.491602 |
| 213948_x_at | Q8N126 | CADM3 | 0.000273 | 0.786682 | 0.016495 | 0.826523 |
| 200982_s_at | P08133 | ANXA6 | 1.69E-05 | 0.727072 | 9.02E-12 | 0.430481 |
| 227434_at | Q6IS24 | WBSCR17 | 2.10E-08 | 0.680663 | 3.22E-09 | 0.578049 |
| 208832_at | Q9UBB4 | ATXN10 | 1.15E-08 | 0.769688 | 7.38E-11 | 0.533159 |
| 208833_s_at | Q9UBB4 | ATXN10 | 6.05E-09 | 0.783255 | 1.66E-10 | 0.453233 |
| 206731_at | Q8WXI2 | CNKSR2 | 1.48E-07 | 0.621315 | 6.62E-08 | 0.480395 |
| 201939_at | Q9NYY3 | PLK2 | 8.50E-08 | 0.640918 | 9.40E-08 | 0.371127 |
| 244194_at | Q9P0K1 | ADAM22 | 6.65E-05 | 0.738454 | 1.40E-05 | 0.799249 |
| 204540_at | Q05639 | EEF1A2 | 2.03E-05 | 0.695973 | 1.38E-10 | 0.399694 |
| 217930_s_at | Q9H0E2 | TOLLIP | 4.11E-08 | 0.625679 | 3.08E-11 | 0.630687 |
| 233881_s_at | Q9H0E2 | TOLLIP | 7.73E-07 | 0.685516 | 8.75E-08 | 0.706724 |
| 213132_s_at | Q8IVS2 | MCAT | 2.66E-07 | 0.786647 | 2.11E-10 | 0.591437 |
| 227154_at | Q96ID5 | IGSF21 | 0.000132 | 0.805047 | 2.37E-09 | 0.594391 |
| 227760_at | Q8WX77 | IGFBPL1 | 5.68E-06 | 0.8123 | 5.89E-06 | 0.70874 |
| 209382_at | Q9BUI4 | POLR3C | 7.44E-07 | 0.810922 | 1.42E-14 | 0.539536 |
| 222849_s_at | Q0VDG4 | SCRN3 | 3.82E-07 | 0.783655 | 0.000512 | 0.638858 |
| 221750_at | Q01581 | HMGCS1 | 7.58E-08 | 0.728292 | 3.93E-06 | 0.568121 |
| 208968_s_at | Q6FI81 | CIAPIN1 | 1.83E-06 | 0.803093 | 1.62E-10 | 0.528623 |
| 208424_s_at | Q6FI81 | CIAPIN1 | 4.78E-06 | 0.809787 | 3.68E-08 | 0.545625 |
| 204945_at | Q16849 | PTPRN | 4.04E-09 | 0.657159 | 0.003139 | 0.727362 |
| 213308_at | Q9UPX8 | SHANK2 | 1.49E-05 | 0.770321 | 0.000234 | 0.649576 |
| 213307_at | Q9UPX8 | SHANK2 | 4.34E-05 | 0.790902 | 0.000509 | 0.725067 |
| 243681_at | Q9UPX8 | SHANK2 | 1.36E-06 | 0.743794 | 0.000901 | 0.621277 |
| 223253_at | Q9UM22 | EPDR1 | 0.003223 | 0.804385 | 6.78E-09 | 0.350698 |
| 236088_at | Q9Y2I2 | NTNG1 | 5.02E-06 | 0.702316 | 6.38E-05 | 0.574203 |
| 209726_at | O75493 | CA11 | 2.68E-06 | 0.784266 | 7.43E-05 | 0.612857 |
| 229506_at | Q5SGD2 | PPM1L | 0.000281 | 0.804209 | 0.006483 | 0.695096 |
| 212217_at | Q4J6C6 | PREPL | 8.47E-05 | 0.745514 | 6.93E-10 | 0.465061 |
| 212216_at | Q4J6C6 | PREPL | 0.000132 | 0.686682 | 1.95E-05 | 0.65304 |
| 214930_at | O94991 | SLITRK5 | 0.000112 | 0.786926 | 3.78E-08 | 0.522078 |
| 204127_at | P40938 | RFC3 | 1.04E-06 | 0.77703 | 0.00063 | 0.689444 |
| 201955_at | P24863 | CCNC | 1.68E-07 | 0.820811 | 0.002272 | 0.71332 |
| 211207_s_at | Q9UKU0 | ACSL6 | 1.70E-05 | 0.750341 | 6.22E-06 | 0.586585 |
| 213683_at | Q9UKU0 | ACSL6 | 0.000966 | 0.795363 | 0.002073 | 0.806969 |
| 203861_s_at | P35609 | ACTN2 | 1.03E-05 | 0.792201 | 3.39E-07 | 0.638016 |
| 223457_at | Q9UBF2 | COPG2 | 0.000181 | 0.822404 | 0.000114 | 0.744039 |
| 224871_at | Q5T0D9 | TPRG1L | 1.80E-05 | 0.825387 | 2.22E-11 | 0.515506 |
| 223530_at | Q9Y2W6 | TDRKH | 9.37E-08 | 0.791504 | 5.48E-10 | 0.65753 |
| 235509_at | Q330K2 | NDUFAF6 | 4.72E-06 | 0.78081 | 1.26E-05 | 0.581988 |
| 205825_at | P29120 | PCSK1 | 0.00019 | 0.535946 | 3.81E-07 | 0.338287 |
| 202154_x_at | Q13509 | TUBB3 | 2.20E-09 | 0.688124 | 2.21E-13 | 0.342618 |
| 213476_x_at | Q13509 | TUBB3 | 3.26E-09 | 0.684574 | 1.80E-12 | 0.323363 |
| 218756_s_at | Q6UWP2 | DHRS11 | 2.42E-07 | 0.677186 | 1.38E-10 | 0.683814 |
| 202078_at | Q9UNS2 | COPS3 | 2.31E-06 | 0.821111 | 2.36E-11 | 0.408127 |
| 1552774_a_at | O95847 | SLC25A27 | 0.000795 | 0.809721 | 7.23E-05 | 0.790442 |
| 1554161_at | O95847 | SLC25A27 | 7.05E-05 | 0.794132 | 0.001865 | 0.746052 |
| 1555385_at | Q00973 | B4GALNT1 | 0.00039 | 0.791186 | 0.000421 | 0.741374 |

## Supplementary Table S2. GO terms enriched by 296 predicted blood-secretory proteins.

| **GO terms of biological processes** | | | |
| --- | --- | --- | --- |
| **GO_Number** | **Term** | **Count** | **P-value** |
| GO:0006468 | protein phosphorylation | 23 | 6.90E-06 |
| GO:0002576 | platelet degranulation | 10 | 5.01E-05 |
| GO:0045669 | positive regulation of osteoblast differentiation | 8 | 5.51E-05 |
| GO:0007017 | microtubule-based process | 6 | 2.87E-04 |
| GO:0048839 | inner ear development | 6 | 5.34E-04 |
| GO:0051090 | regulation of sequence-specific DNA binding transcription factor activity | 5 | 6.88E-04 |
| GO:0023014 | signal transduction by protein phosphorylation | 6 | 7.44E-04 |
| GO:0007417 | central nervous system development | 9 | 8.27E-04 |
| GO:0010862 | positive regulation of pathway-restricted SMAD protein phosphorylation | 6 | 0.001112 |
| GO:0007258 | JUN phosphorylation | 3 | 0.00158 |
| GO:0060292 | long term synaptic depression | 4 | 0.002099 |
| GO:0030501 | positive regulation of bone mineralization | 5 | 0.002504 |
| GO:0032876 | negative regulation of DNA endoreduplication | 3 | 0.002605 |
| GO:0050714 | positive regulation of protein secretion | 5 | 0.002781 |
| GO:0006950 | response to stress | 6 | 0.003249 |
| GO:0060291 | long-term synaptic potentiation | 5 | 0.003397 |
| GO:0001676 | long-chain fatty acid metabolic process | 4 | 0.004692 |
| GO:0009725 | response to hormone | 5 | 0.004893 |
| GO:0002315 | marginal zone B cell differentiation | 3 | 0.005353 |
| GO:0008152 | metabolic process | 9 | 0.006691 |
| GO:0070262 | peptidyl-serine dephosphorylation | 3 | 0.00706 |
| GO:0038095 | Fc-epsilon receptor signaling pathway | 9 | 0.009338 |
| GO:0001558 | regulation of cell growth | 6 | 0.010216 |
| GO:0008284 | positive regulation of cell proliferation | 16 | 0.010486 |
| GO:0007368 | determination of left/right symmetry | 5 | 0.012635 |
| GO:0001654 | eye development | 4 | 0.01285 |
| GO:0001822 | kidney development | 6 | 0.013678 |
| GO:0044319 | wound healing, spreading of cells | 3 | 0.015936 |
| GO:0015908 | fatty acid transport | 3 | 0.015936 |
| GO:0050777 | negative regulation of immune response | 3 | 0.015936 |
| GO:0045747 | positive regulation of Notch signaling pathway | 4 | 0.016658 |
| GO:0043123 | positive regulation of I-kappaB kinase/NF-kappaB signaling | 8 | 0.017233 |
| GO:0018105 | peptidyl-serine phosphorylation | 7 | 0.017254 |
| GO:0060391 | positive regulation of SMAD protein import into nucleus | 3 | 0.018632 |
| GO:0043066 | negative regulation of apoptotic process | 15 | 0.01866 |
| GO:0007613 | memory | 5 | 0.018914 |
| GO:0008584 | male gonad development | 6 | 0.019415 |
| GO:0030889 | negative regulation of B cell proliferation | 3 | 0.021504 |
| GO:0060347 | heart trabecula formation | 3 | 0.021504 |
| GO:0001666 | response to hypoxia | 8 | 0.023803 |
| GO:0018107 | peptidyl-threonine phosphorylation | 4 | 0.024263 |
| GO:0060397 | JAK-STAT cascade involved in growth hormone signaling pathway | 3 | 0.024547 |
| GO:0010468 | regulation of gene expression | 6 | 0.024623 |
| GO:0001501 | skeletal system development | 7 | 0.025751 |
| GO:0000086 | G2/M transition of mitotic cell cycle | 7 | 0.025751 |
| GO:0030199 | collagen fibril organization | 4 | 0.025974 |
| GO:0007165 | signal transduction | 29 | 0.027091 |
| GO:0003222 | ventricular trabecula myocardium morphogenesis | 3 | 0.027753 |
| GO:0032924 | activin receptor signaling pathway | 3 | 0.027753 |
| GO:0050708 | regulation of protein secretion | 3 | 0.027753 |
| GO:0071300 | cellular response to retinoic acid | 5 | 0.028106 |
| GO:0042325 | regulation of phosphorylation | 3 | 0.031118 |
| GO:0001824 | blastocyst development | 3 | 0.031118 |
| GO:0009416 | response to light stimulus | 3 | 0.031118 |
| GO:0042704 | uterine wall breakdown | 2 | 0.032604 |
| GO:1905075 | positive regulation of occluding junction disassembly | 2 | 0.032604 |
| GO:0045216 | cell-cell junction organization | 3 | 0.034635 |
| GO:0022617 | extracellular matrix disassembly | 5 | 0.036476 |
| GO:0060021 | palate development | 5 | 0.036476 |
| GO:0051493 | regulation of cytoskeleton organization | 3 | 0.038297 |
| GO:0006486 | protein glycosylation | 6 | 0.038773 |
| GO:0045766 | positive regulation of angiogenesis | 6 | 0.041312 |
| GO:0006541 | glutamine metabolic process | 3 | 0.0421 |
| GO:0030198 | extracellular matrix organization | 8 | 0.043916 |
| GO:0034447 | very-low-density lipoprotein particle clearance | 2 | 0.048506 |
| GO:1902731 | negative regulation of chondrocyte proliferation | 2 | 0.048506 |
|  | **GO terms of cellular components** |  |  |
| **GO_Number** | **Term** | **Count** | **P-value** |
| GO:0005578 | proteinaceous extracellular matrix | 19 | 2.57E-07 |
| GO:0005739 | mitochondrion | 42 | 2.53E-05 |
| GO:0005829 | cytosol | 81 | 2.81E-05 |
| GO:0043025 | neuronal cell body | 17 | 4.07E-05 |
| GO:0070062 | extracellular exosome | 69 | 1.30E-04 |
| GO:0005576 | extracellular region | 43 | 7.45E-04 |
| GO:0045121 | membrane raft | 11 | 0.001588139 |
| GO:0048471 | perinuclear region of cytoplasm | 21 | 0.002004013 |
| GO:0031012 | extracellular matrix | 13 | 0.002598299 |
| GO:0030133 | transport vesicle | 7 | 0.00383716 |
| GO:0043235 | receptor complex | 8 | 0.003930548 |
| GO:0016020 | membrane | 51 | 0.004424892 |
| GO:0030864 | cortical actin cytoskeleton | 5 | 0.004531117 |
| GO:0005856 | cytoskeleton | 14 | 0.006031973 |
| GO:0030141 | secretory granule | 6 | 0.006132434 |
| GO:1990712 | HFE-transferrin receptor complex | 3 | 0.006456186 |
| GO:0005604 | basement membrane | 6 | 0.008055948 |
| GO:0000794 | condensed nuclear chromosome | 4 | 0.00937482 |
| GO:0031093 | platelet alpha granule lumen | 5 | 0.010820126 |
| GO:0005615 | extracellular space | 33 | 0.012301208 |
| GO:0030424 | axon | 9 | 0.024299166 |
| GO:0005874 | microtubule | 11 | 0.025532981 |
| GO:0031143 | pseudopodium | 3 | 0.028579837 |
| GO:0008280 | cohesin core heterodimer | 2 | 0.031141741 |
| GO:0043209 | myelin sheath | 7 | 0.03294599 |
| GO:0005737 | cytoplasm | 97 | 0.038533572 |
| GO:0000159 | protein phosphatase type 2A complex | 3 | 0.03872124 |
| GO:0030425 | dendrite | 11 | 0.039487961 |
| GO:0043202 | lysosomal lumen | 5 | 0.044782566 |
| GO:0005925 | focal adhesion | 12 | 0.045145107 |
|  | **GO terms of molecular functions** |  |  |
| **GO_Number** | **Term** | **Count** | **P-value** |
| GO:0005524 | ATP binding | 46 | 6.95E-05 |
| GO:0004675 | transmembrane receptor protein serine/threonine kinase activity | 4 | 0.000356 |
| GO:0004705 | JUN kinase activity | 3 | 0.000819 |
| GO:0019888 | protein phosphatase regulator activity | 5 | 0.001286 |
| GO:0004707 | MAP kinase activity | 4 | 0.00145 |
| GO:0005178 | integrin binding | 8 | 0.001866 |
| GO:0005200 | structural constituent of cytoskeleton | 8 | 0.002433 |
| GO:0004722 | protein serine/threonine phosphatase activity | 6 | 0.002751 |
| GO:0016757 | transferase activity, transferring glycosyl groups | 6 | 0.003431 |
| GO:0004674 | protein serine/threonine kinase activity | 15 | 0.004343 |
| GO:0005509 | calcium ion binding | 23 | 0.004404 |
| GO:0005201 | extracellular matrix structural constituent | 6 | 0.005138 |
| GO:0008201 | heparin binding | 9 | 0.005404 |
| GO:0005114 | type II transforming growth factor beta receptor binding | 3 | 0.005487 |
| GO:0008092 | cytoskeletal protein binding | 5 | 0.008232 |
| GO:0004702 | receptor signaling protein serine/threonine kinase activity | 5 | 0.011615 |
| GO:0004859 | phospholipase inhibitor activity | 3 | 0.013753 |
| GO:0003924 | GTPase activity | 10 | 0.016737 |
| GO:0003725 | double-stranded RNA binding | 5 | 0.018681 |
| GO:0004467 | long-chain fatty acid-CoA ligase activity | 3 | 0.019082 |
| GO:0016740 | transferase activity | 6 | 0.022106 |
| GO:0042802 | identical protein binding | 21 | 0.02583 |
| GO:0046332 | SMAD binding | 4 | 0.034533 |
| GO:0005112 | Notch binding | 3 | 0.035447 |
| GO:0005520 | insulin-like growth factor binding | 3 | 0.035447 |
| GO:0003824 | catalytic activity | 8 | 0.038543 |
| GO:0042288 | MHC class I protein binding | 3 | 0.03919 |
| GO:0004653 | polypeptide N-acetylgalactosaminyltransferase activity | 3 | 0.043076 |
| GO:0008601 | protein phosphatase type 2A regulator activity | 3 | 0.047099 |
| GO:0070008 | serine-type exopeptidase activity | 2 | 0.049114 |
